# The comorbidity burden of type 2 diabetes mellitus: patterns, clusters and predictions from a large English primary care cohort

**DOI:** 10.1186/s12916-019-1373-y

**Published:** 2019-07-25

**Authors:** Magdalena Nowakowska, Salwa S. Zghebi, Darren M. Ashcroft, Iain Buchan, Carolyn Chew-Graham, Tim Holt, Christian Mallen, Harm Van Marwijk, Niels Peek, Rafael Perera-Salazar, David Reeves, Martin K. Rutter, Stephen F. Weng, Nadeem Qureshi, Mamas A. Mamas, Evangelos Kontopantelis

**Affiliations:** 1 NIHR School for Primary Care Research, Centre for Primary Care and Health Services Research, Manchester Academic Health Science Centre (MAHSC), University of Manchester, 5th floor Williamson Building, Manchester, M13 9PL UK; 2 Division of Population Health, Health Services Research & Primary Care, School of Health Sciences, Faculty of Biology, Medicine and Health, Manchester Academic Health Science Centre (MAHSC), University of Manchester, 5th floor Williamson Building, Manchester, M13 9PL UK; 3 Division of Pharmacy and Optometry, School of Health Sciences, Faculty of Biology, Medicine and Health, Manchester Academic Health Science Centre (MAHSC), University of Manchester, Manchester, M13 9PL UK; 4 NIHR Greater Manchester Patient Safety Translational Research Centre, University of Manchester, Manchester, M13 9PL UK; 5 Division of Informatics, Imaging, and Data Science, School of Health Sciences, Faculty of Biology, Medicine and Health, Manchester Academic Health Science Centre (MAHSC), University of Manchester, Manchester, M13 9PL UK; 6 Department of Public Health and Policy, Institute of Population Health Sciences, University of Liverpool, Liverpool, L69 3BX UK; 7 Research Institute for Primary Care and Health Sciences, Faculty of Medicine and Health Sciences, Keele University, DJW 1.54a, Staffordshire, ST5 5BJ UK; 8 Nuffield Department of Primary Care Health Sciences, University of Oxford, Oxford, OX2 6GG UK; 9 Division of Primary Care and Public Health, Brighton and Sussex Medical School, University of Sussex, Brighton, BN1 9PH UK; 10 NIHR Manchester Biomedical Research Centre, Manchester Academic Health Science Centre (MAHSC), University of Manchester, Manchester, M13 9PL UK; 11 Centre for Biostatistics, Division of Population Health, Health Services Research and Primary Care, School of Health Sciences, Faculty of Biology, Medicine and Health, University of Manchester, Manchester, M13 9PL UK; 12 Division of Diabetes, Endocrinology and Gastroenterology, Faculty of Medicine, Biology and Health, University of Manchester, Manchester, M13 9PL UK; 13 Manchester Diabetes Centre, Manchester Academic Health Science Centre, Manchester University NHS Foundation Trust, Manchester, M13 0JE UK; 14 Primary Care Stratified Medicine (PRISM), Division of Primary Care, School of Medicine, University of Nottingham, Nottingham, NG7 2RD UK; 15 Keele Cardiovascular Research Group, Centre for Prognosis Research, Institute for Primary Care and Health Sciences, Keele University, Stoke-on-Trent, ST4 7QB UK

**Keywords:** Comorbidity, Type 2 diabetes mellitus, CPRD, Prevalence, Primary care

## Abstract

**Background:**

The presence of additional chronic conditions has a significant impact on the treatment and management of type 2 diabetes (T2DM). Little is known about the patterns of comorbidities in this population. The aims of this study are to quantify comorbidity patterns in people with T2DM, to estimate the prevalence of six chronic conditions in 2027 and to identify clusters of similar conditions.

**Methods:**

We used the Clinical Practice Research Datalink (CPRD) linked with the Index of Multiple Deprivation (IMD) data to identify patients diagnosed with T2DM between 2007 and 2017. 102,394 people met the study inclusion criteria. We calculated the crude and age-standardised prevalence of 18 chronic conditions present at and after the T2DM diagnosis. We analysed longitudinally the 6 most common conditions and forecasted their prevalence in 2027 using linear regression. We used agglomerative hierarchical clustering to identify comorbidity clusters. These analyses were repeated on subgroups stratified by gender and deprivation.

**Results:**

More people living in the most deprived areas had ≥ 1 comorbidities present at the time of diagnosis (72% of females; 64% of males) compared to the most affluent areas (67% of females; 59% of males). Depression prevalence increased in all strata and was more common in the most deprived areas. Depression was predicted to affect 33% of females and 15% of males diagnosed with T2DM in 2027. Moderate clustering tendencies were observed, with concordant conditions grouped together and some variations between groups of different demographics.

**Conclusions:**

Comorbidities are common in this population, and high between-patient variability in comorbidity patterns emphasises the need for patient-centred healthcare. Mental health is a growing concern, and there is a need for interventions that target both physical and mental health in this population.

**Electronic supplementary material:**

The online version of this article (10.1186/s12916-019-1373-y) contains supplementary material, which is available to authorized users.

## Background

The prevalence of type 2 diabetes (T2DM) is increasing in the UK and internationally. Diabetes (all types) is estimated to affect 1 in 11 adults aged 20 to 79 years, or 415 million adults globally [1]. In 2016, it was the seventh leading cause of death worldwide with an estimated 1.6 million deaths directly caused by diabetes [2]. In the UK over 90% of diabetes cases are type 2 diabetes [3], with most individuals having at least one other chronic condition [4]. Diabetes-related healthcare outcomes, treatment options, care needs and associated cost are complicated by the presence of comorbidities—chronic conditions existing in addition to T2DM.

Due to similar risk factors, such as obesity, endothelial dysfunction, vascular inflammation and dyslipidaemia [5], people with T2DM have higher risks of cardiovascular complications [6], end-stage renal disease [7] and hypertension [8]. However, individuals with T2DM have also been found to have higher risks of depression [9], thyroid gland diseases [10] and chronic obstructive pulmonary disease (COPD) [11]. People with multiple chronic conditions report a number of barriers to self-care such as physical limitations, lack of knowledge, financial constraints, logistics of obtaining care and the need for social and emotional support [12]. The specific combination of comorbidities in diabetes (type 1 and 2) patients has been found to impact their ability to prioritise and manage the disease [13]. Patients with conditions considered unrelated to diabetes may need additional support in making decisions about care priorities and self-management activities [13]. While the presence of diabetes-“concordant” conditions (i.e. sharing the same management goals), tends to be positively associated with quality of care [14], certain “discordant” comorbidities, like depression and arthritis, impact on treatment options, posing barriers to lifestyle changes and self-care behaviours recommended for diabetes management [14–16].

The specific combinations of conditions present dictate the needs of patients, management priorities and the associated demand on healthcare services [17]. A better understanding of the nature, prevalence and patterns of comorbidities in T2DM patients may provide key insights for managing patients with multiple conditions in primary care and facilitate a more patient-centred approach in risk assessment and more appropriate and tailored therapeutic interventions. Understanding and forecasting the prevalence of specific comorbidities can inform policy-makers in planning and structuring health services to meet the future demands of the population.

In this study, we explored the comorbidities’ patterns occurring in patients with T2DM over time, as seen in English primary care. We quantified the prevalence of 18, highly prevalent and well-recorded physical and mental health conditions and compared the patterns in subgroups of patients stratified by gender, age and socioeconomic deprivation. Focusing on an incidental cohort of patients with T2DM, we explored the patterns in comorbidity occurrence at the time of T2DM diagnosis and after 2, 5and 9 years of follow-up.

## Methods

### Data source

The Clinical Practice Research Datalink (CPRD) is a database of anonymised electronic, primary health records. In January 2017, the CPRD held data on nearly 17 million active and historical patients registered with 714 general practices across the UK. It contains information on diagnoses, referrals, tests and therapy records, which are mainly recorded using Read clinical codes. Additional data is available for a subset of English practices (nearly 75% of English practices; 58% of all UK CPRD practices) which consented to participate in the CPRD linkage scheme and provided patient-level information. To obtain information on social deprivation at the level of the patient’s postcode, we used the linked information on the quintiles from the 2015 Index of Multiple Deprivation (IMD) measure, which aggregates data on income, employment, health and disability, education and training, barriers to housing and services, crime and living environment.

### Study sample

People registered with a general practice in England meeting CPRD data quality standards and with the first T2DM Read code recorded at any point between 1 April 2007 and 31 March 2017 were included. The inclusion criteria for this study were as follows: patient registered with a CPRD practice for at least 365 days before T2DM diagnosis, aged 35 years and older and no recorded diagnostic code for type-1 diabetes mellitus. In the UK, T2DM has been incentivised since 2004 through a national pay-for-performance scheme, the Quality and Outcomes Framework (QOF), along with another 20 clinical domains approximately, resulting in uniformity in Read code usage and recording. The index date was defined as the date of first recorded code for T2DM and the follow-up as the time between the index date and the earliest of date of death, transfer out of practice date and last date of data collection from the practice or the end of study period (31 March 2017). The lists of codes used to establish the presence of each comorbidity were downloaded from clinicalcodes.org and CPRD@Cambridge websites.

### Defining comorbidities

We selected the following 18 conditions: coronary heart disease (CHD), chronic kidney disease (CKD), atrial fibrillation, stroke, hypertension, heart failure, peripheral vascular disease (PVD), rheumatoid arthritis, cancer, osteoporosis, depression, asthma, chronic obstructive pulmonary disease (COPD), dementia, severe mental illness (SMI), epilepsy, hypothyroidism and learning disability. The reporting of these conditions is financially incentivised under the QOF, and consequently, they are well-recorded in the CPRD. The presence of asthma, epilepsy and depression was determined using Read codes and prescription data, since these can be acute or resolvable. Each condition was considered to be present at the index date if it satisfied the definition criteria at the time of the T2DM diagnosis (Additional file 1: Table S1). Each condition was considered to be present during the follow-up period if it satisfied the definition criteria at the index date or at any time during the follow-up.

### Statistical analysis

First, we used the sample in terms of the total number of comorbidities present at the index date and after 1 year, 5 years and 9 years of follow-up. We examined the total number of comorbidities present at and after the index date, stratified by gender and social deprivation quintiles. Age-standardised prevalence was calculated using the direct age standardisation to the 2013 European Standard Population using 5-year age bands up to 95+ years old. Differences between means of categorical variables were tested using 2-sample *t* tests.

We calculated the age-standardised prevalence of each condition, stratified by gender, for patients from the least and most deprived areas. We also calculated the crude and age-standardised co-prevalence of each pair of comorbidities for the whole sample and stratified by gender, deprivation (least and most deprived areas) and age (using 35–54-, 55–74- and 75+-year-old age bands).

We longitudinally calculated the prevalence of each comorbidity present at the time of the T2DM diagnosis in the incidental cohort of patients with T2DM, for financial years (April to March) 2007/2008 to 2016/2017. To forecast the proportion of people diagnosed with T2DM in the next 10 years that will also have a particular comorbidity present at the time of diagnosis, we used linear regression on log-transformed, age-standardised prevalence. For clarity of results, we present the patterns for the six most prevalent conditions as the prevalence of remaining conditions remained relatively low and stable over the study period.

Lastly, we selected patients with two or more comorbidities present at the index date and used agglomerative hierarchical clustering to identify groups of similar conditions. Similarity was assessed using the tetrachoric correlation coefficient. Tetrachoric correlation estimates what the correlation for two binary variables would be if they were measured on a continuous scale. We used Ward’s linkage method to group conditions. At each linkage step, Ward’s method finds a pair of clusters that leads to a minimum increase in total within-cluster variance after merging. To avoid chaining (low prevalence comorbidities being sequentially linked to existing clusters), we excluded conditions with prevalence in a given group below 3%. Cluster analysis was stratified by gender, age bands (35 to 54 years, 55 to 74 years and ≥ 75 years old) and deprivation using the least and most deprived quintiles. We present the results for the whole sample. Stratified results are available in Additional file 1: Figure S12–S18. To assess the progression in clustering patterns, we performed the cluster analysis for conditions present at the time of T2DM diagnosis and those present at 2, 5 and 9 years after. We plotted the results in dendrograms and identified clusters using visual analysis. Dendrograms visually represent the clustering. The heights at which conditions fuse together correspond to their similarity. The earlier the branches merge, the more similar the groups of conditions are. The clustering structure was measured using the agglomerative coefficient, with values closer to zero suggesting tight clustering of objects and values closer to one suggesting less well-formed clusters. Due to differences in sample sizes, agglomerative coefficients should not be compared across groups. We used R version 3.4.2 for the analysis and data preparation.

## Results

**Fig. 1:**
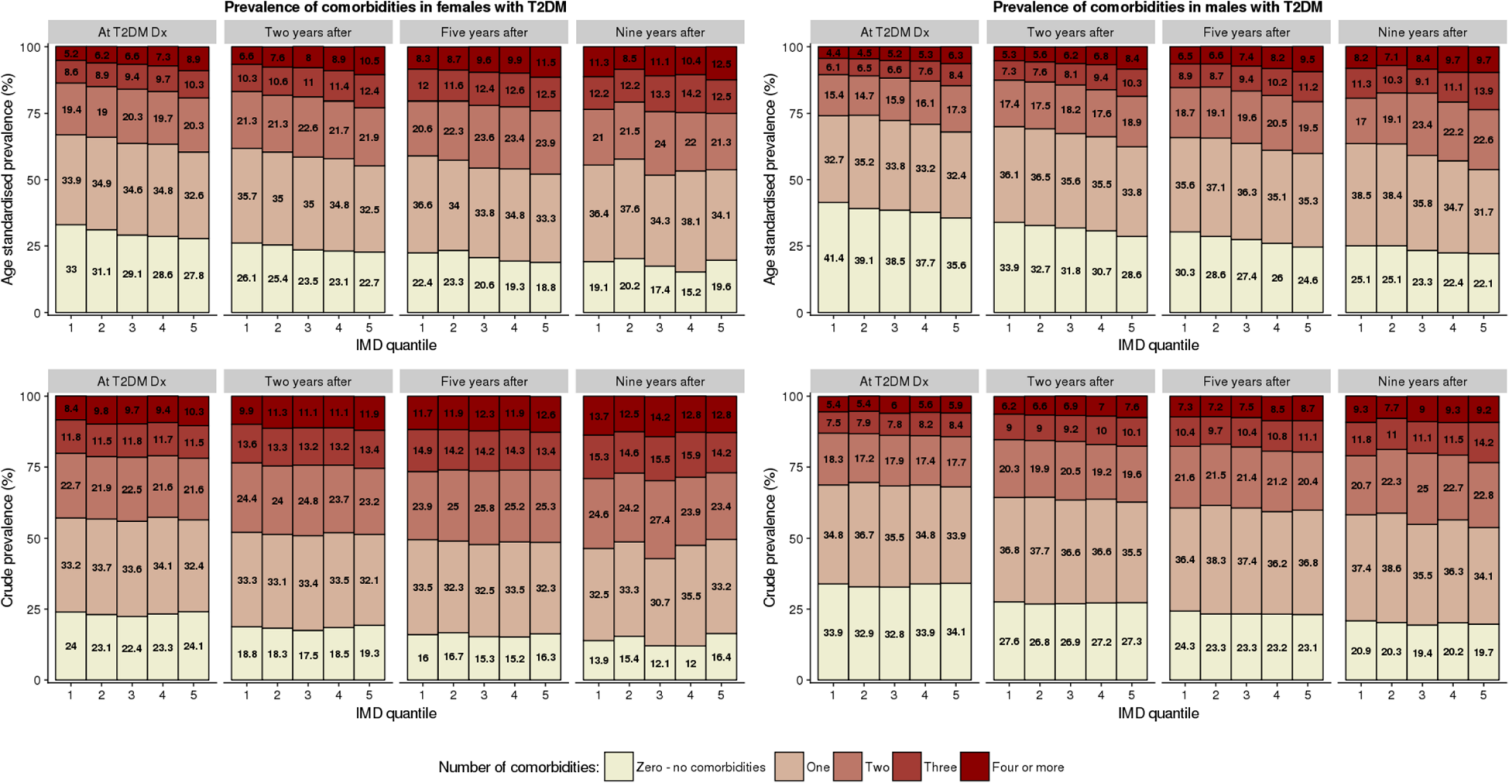
Age-standardised and crude prevalence of multiple conditions in patients with T2DM. Age-standardised (top) and crude (bottom) prevalence of zero, one, two, three and four or more comorbidities present in patients with type 2 diabetes at the time of type 2 diabetes diagnosis and after 2, 5 and 9 years of follow-up. Stratified by gender and deprivation. T2DM - type 2 diabetes mellitus; Dx - diagnosis; IMD - Index of Multiple Deprivation

We identified 102,394 people with incident T2DM during the study period, who met the study inclusion criteria. A flow chart of the data cleaning process is available in Additional file 1: Figure S1. The median (LQ, 25th centile; UQ, 75th centile) follow-up was 4.9 years (LQ, 2.8; UQ, 7.3). Over half of the sample (56.3%) was male with an average (mean ± standard deviation) age at diagnosis of 60.3 (± 12.5) (Table 1). On average, women were diagnosed at an older age (63.7 ± 13.6, *p* < 0.001) and had more comorbidities at the time of T2DM diagnosis compared to males (1.6 ± 1.4 vs 1.2 ± 1.2, *p* < 0.001). People from the most deprived areas were diagnosed with T2DM at a younger age, compared to those from the most affluent areas (59.3 ± 13 vs 63.9 ± 12.8, *p* < 0.001). The age-standardised prevalence of one or more comorbid conditions was 33.3% (95% confidence interval: 32.5%; 34.1%) for the least deprived areas and 32.7% (31.7%; 33.3%) for the most deprived areas (Fig. 1). For four or more comorbid conditions, the age-standardised prevalence was 2.9% (2.7%; 3.1%) in the most affluent areas and 4.4% (4.1%; 4.7%) in the most deprived areas. In all subgroups (by sex and deprivation), the proportion of people with zero comorbidities decreased during the follow-up period (Fig. 1).

**Table 1:** Descriptive statistics on patients with type 2 diabetes mellitus (type 2 diabetes) and additional comorbidity

	*N* (%)	Age (mean ± SD)	Follow-up period (median (LQ; UQ))	Number of comorbidities at T2DM diagnosis (mean ± SD)	Number of comorbidities 2 years after T2DM diagnosis (mean ± SD) (sample surviving 2 years)	Number of comorbidities 5 years after T2DM diagnosis (mean ± SD) (sample surviving 5 years)	Number of comorbidities 9 years after T2DM diagnosis (mean ± SD) (sample surviving 9 years)
Total cohort	102,394 (100)	62.1 ± 13.1	4.9 (2.8; 7.3)	1.4 ± 1.3	1.5 ± 1.4 (84,350)	1.6 ± 1.4 (50,475)	1.7 ± 1.4 (8977)
Gender							
Females	44,764 (43.7)	63.7 ± 13.6	4.9 (2.7; 7.3)	1.6 ± 1.4	1.7 ± 1.4 (36,669)	1.8 ± 1.4 (21,830)	1.9 ± 1.5 (3942)
Males	57,630 (56.3)	60.7 ± 12.5	5 (2.8; 7.3)	1.2 ± 1.2	1.4 ± 1.3 (47,681)	1.5 ± 1.3 (28,645)	1.6 ± 1.4 (5035)
Age bands							
35–54 years	31,545 (30.8)	46.8 ± 5.2	5.1 (2.9; 7.4)	0.8 ± 0.9	0.9 ± 1 (26,368)	1 ± 1 (16,106)	1.1 ± 1 (2893)
55–74 years	51,288 (50.1)	64.2 ± 5.6	5.2 (3; 7.5)	1.4 ± 1.2	1.6 ± 1.3 (42,950)	1.7 ± 1.3 (26,618)	1.9 ± 1.4 (4871)
75+ years	19,561 (19.1)	81 ± 4.9	4.1 (2.1; 6.5)	2.3 ± 1.6	2.5 ± 1.6 (15,032)	2.6 ± 1.6 (7751)	2.8 ± 1.5 (1213)
IMD quintiles							
Quintile 1—least deprived	19,110 (18.7)	63.9 ± 12.8	5 (2.8; 7.3)	1.3 ± 1.3	1.5 ± 1.3 (15,756)	1.6 ± 1.4 (9574)	1.7 ± 1.4 (1682)
Quintile 2	20,722 (20.2)	63.4 ± 13	5.1 (2.8; 7.4)	1.4 ± 1.3	1.5 ± 1.3 (17,223)	1.6 ± 1.4 (10,500)	1.7 ± 1.4 (1878)
Quintile 3	21,572 (21.1)	62.7 ± 13	4.9 (2.8; 7.3)	1.4 ± 1.3	1.5 ± 1.3 (17,811)	1.6 ± 1.4 (10,605)	1.8 ± 1.4 (1884)
Quintile 4	21,393 (20.9)	61 ± 13.2	4.9 (2.7; 7.2)	1.4 ± 1.3	1.5 ± 1.4 (17,489)	1.7 ± 1.4 (10,334)	1.7 ± 1.4 (1839)
Quintile 5—most deprived	19,597 (19.1)	59.3 ± 13	4.8 (2.7; 7.3)	1.4 ± 1.4	1.6 ± 1.4 (16,071)	1.7 ± 1.4 (9462)	1.7 ± 1.4 (1694)

**Fig. 2:**
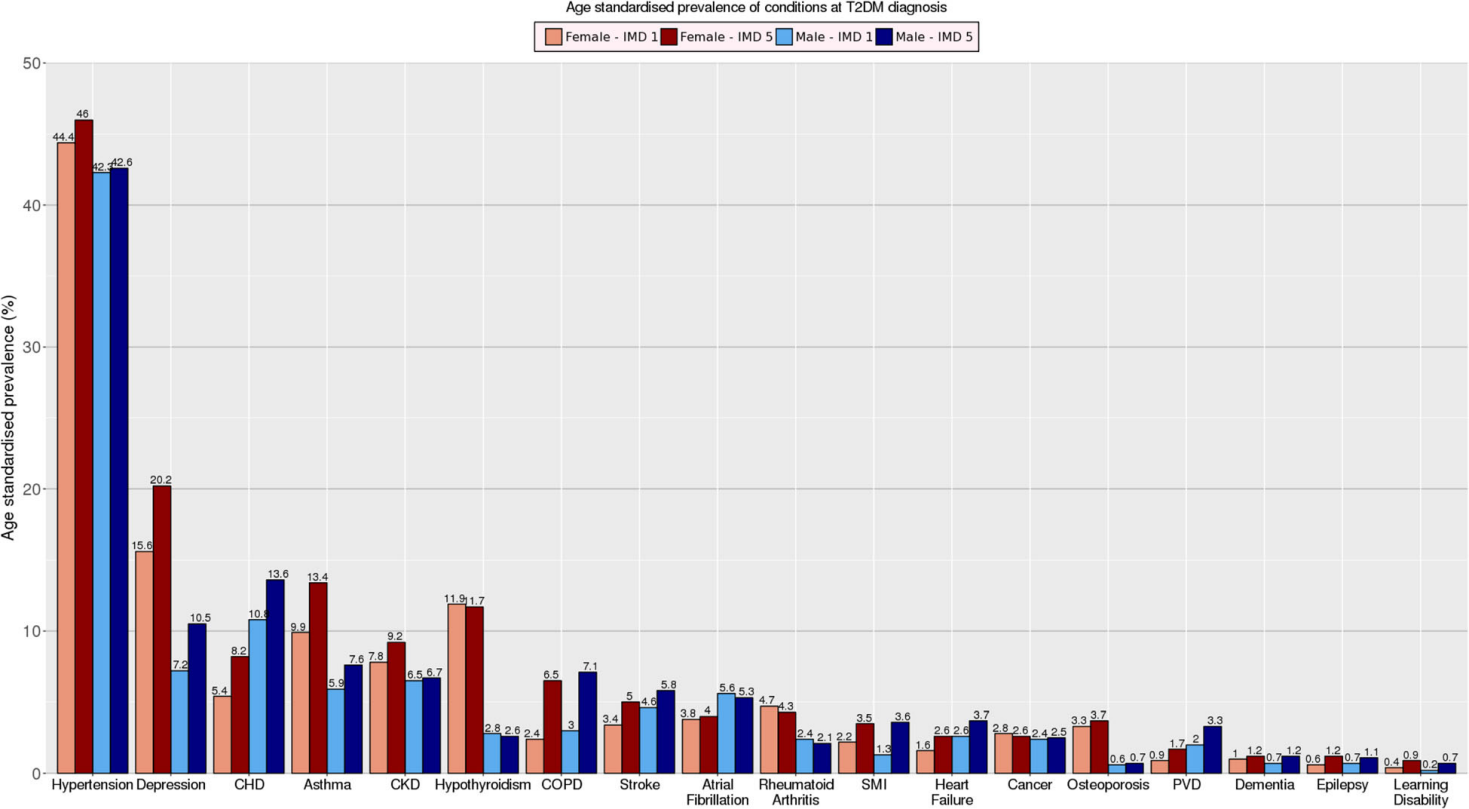
Age-adjusted prevalence of chronic conditions among patients with T2DM. Age-adjusted prevalence of chronic conditions among females and males with type 2 diabetes from the least and most deprived areas at the time of type 2 diabetes diagnosis. IMD - Index of Multiple Deprivation; CHD - coronary heart disease; CKD - chronic kidney disease; COPD - chronic obstructive pulmonary disease; PVD - peripheral vascular disease; SMI - severe mental illness

Hypertension was the most common condition among all patients, with higher prevalence among females than males (42.8% [42.3–43.3%] vs 45.8% [45%; 46.4%]) (Fig. 2, crude prevalence Additional file 1: Figure S2). In females, the second most prevalent condition was depression, with higher prevalence in females from the most deprived areas (20.2% [19.3%; 21.1%]), than from most affluent areas (15.6% [14.7%; 16.5%]). In males, the second most prevalent condition was CHD with higher prevalence among males from the most deprived areas (13.6% [12.9%; 14.3%]), than from the most affluent areas (10.8% [10.3%; 11.3%]). During follow-up, the prevalence of depression and asthma decreased in all groups whereas the prevalence of all other conditions’ increased (prevalence rates for SMI, dementia, epilepsy and learning disability was too low to make meaningful comparisons) (Additional file 1: Figure S3). Hypertension and CKD had the highest age-standardised co-prevalence rate among all patients, at 12.1% at the time of T2DM diagnosis and 15.4%, 17.8% and 21.5% after 2, 5 and 9 years from the T2DM diagnosis (Additional file 1: Figure S4–S11).

**Fig. 3:**
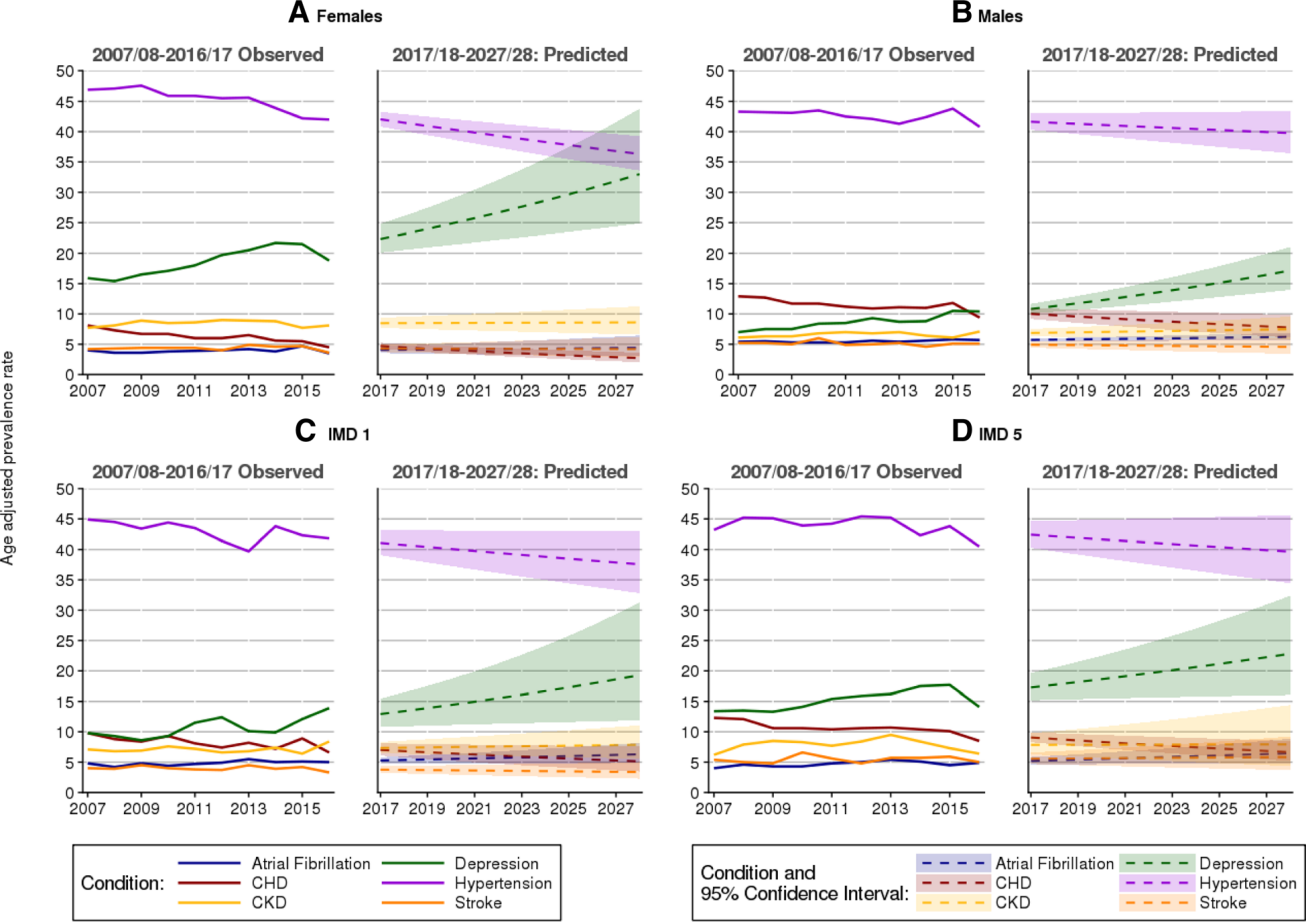
Observed and predicted prevalence of selected conditions in patients with T2DM. Observed and predicted prevalence of selected conditions present at the time of type 2 diabetes mellitus (type 2 diabetes) diagnosis stratified by gender (**a**, **b**) and deprivation (**c**, **d**). IMD - Index of Multiple Deprivation; CHD - coronary heart disease; CKD - chronic kidney disease; COPD - chronic obstructive pulmonary disease; PVD - peripheral vascular disease; SMI - severe mental illness

Our longitudinal analysis showed a steady decrease in the prevalence of hypertension and relatively stable prevalence rates for CHD, CKD, stroke and atrial fibrillation (Fig. 3). The prevalence of depression increased during the study period for all analysed groups. In females, the age-standardised prevalence rate of depression increased from 15.9% (95% CI 14.8%; 17.0%) in 2007 to 21.5% (19.7%; 20.8%) in 2015 and 18.8% (16.8%; 20.8%) in 2016. In males, the age-standardised prevalence rate of depression increased from 7.0% (3.4%; 7.6%) in 2007 to 10.4% (9.1%; 11.7%) in 2016. If the current trend continues, depression can affect over a third of females diagnosed with T2DM by 2026 (age-standardised prevalence, 30.7% [23.9%; 39.4%]) and over 15% (13.2%; 18.9%) of males. The prevalence of depression increased from 9.8% (8.5%; 11.1%) in 2007 to 14.9% (11.3%; 16.5%) in 2016 in the most affluent areas. In the most deprived areas, it increased13.4% (12.0%; 14.8%) in 2007 to 17.7% (15.3%; 19.6%) in 2015 and to 14.1% (11.5%; 16.7%) in 2016. If current trend continues, depression is predicted to affect 17.9% (11.7%; 27.5%) of people in the most affluent and 21% (15.9%; 29.5%) of people from the most deprived areas by 2026.

**Fig. 4:**
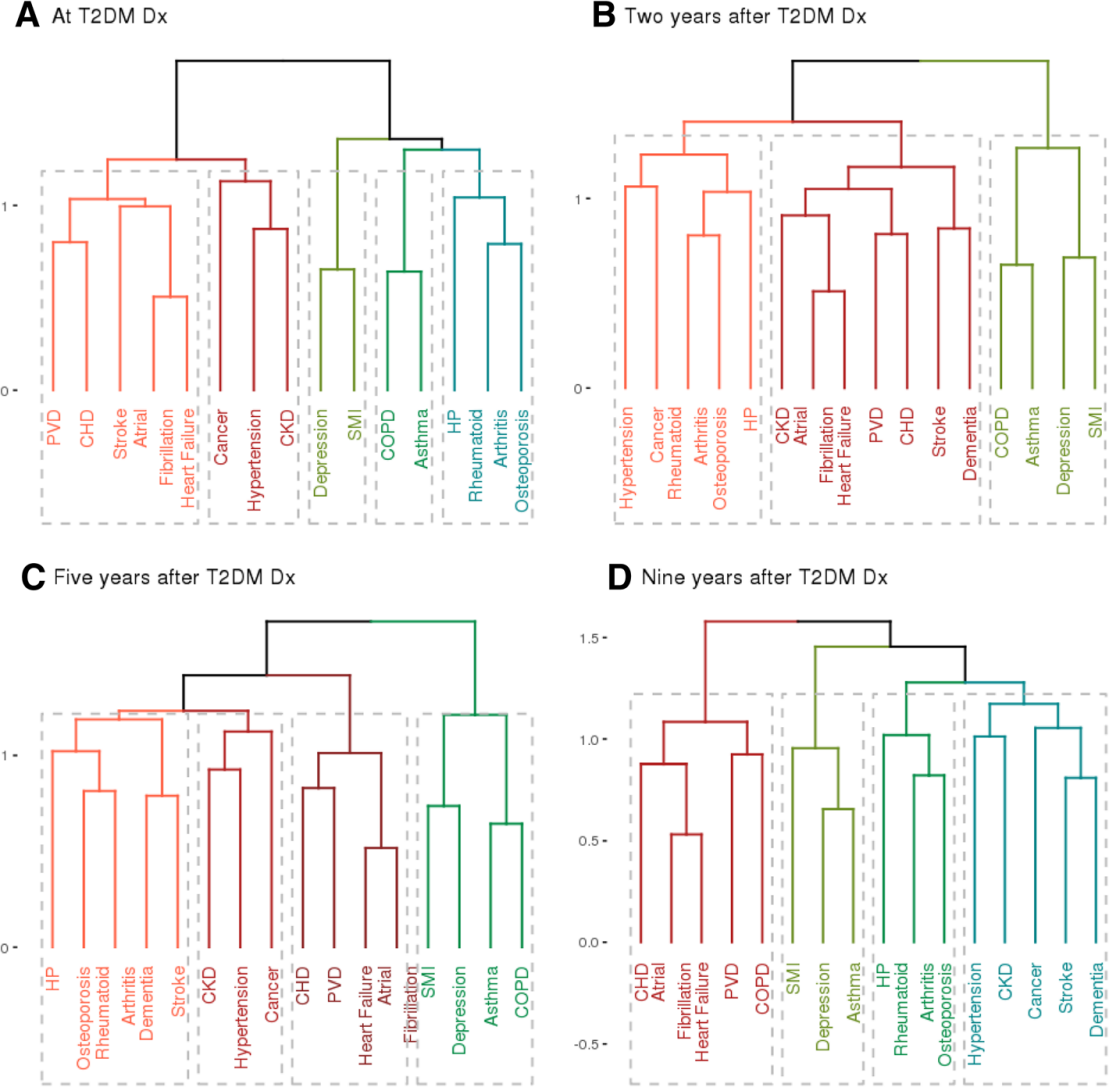
Cluster analysis of comorbidities in people with type 2 diabetes. Cluster analysis of comorbidities in people with type 2 diabetes at the time of the diagnosis (**a**), 2 (**b**), 5 (**c**) and 9 (**d**) years after. CHD - coronary heart disease; CKD - chronic kidney disease; COPD - chronic obstructive pulmonary disease; HP - hypothyroidism; PVD - peripheral vascular disease; SMI - severe mental illness

The hierarchical cluster analysis showed conditions being grouped into two main clusters: the first composed of atrial fibrillation, heart failure, PVD, CHD, cancer, stroke, hypertension and CKD and the second composed of depression, SMI, COPD, asthma, hypothyroidism, rheumatoid arthritis and osteoporosis (Fig. 4). This pattern was similar in all analysed groups with cancer being included in the first cluster for males, people from the most deprived areas, people age 35 to 74 and 75 and over (Additional file 1: Figure S12–S18). However, cancer was linked with cluster two in females, people from the least deprived areas and people age 55–74. Moderate clustering tendencies have been observed for conditions present at the time of T2DM diagnosis with the agglomerative coefficient around 0.45 with some variations between groups.

## Discussion

### Summary

We showed important changes in the comorbidity patterns in a large real-world cohort of people living with T2DM, using data from the UK primary care. Our findings are relevant to patients, clinicians and policy-makers and can inform on the healthcare needs and how best to prioritise and deliver care for people with T2DM. We identified alarming levels and trends of depression prevalence, which we estimated will continue to grow over the next decade. This could have major consequences for how to offer these patients integrated care. Health systems will have to respond to a growing need for diagnosis and management of mental health problems among people with T2DM, underpinned with established links between depression and poor glycaemic control [18], treatment adherence [19], diabetes complications [9] and mortality [20]. The differences in comorbidity patterns observed in groups stratified by gender and social deprivation highlight the need to address the present and increasing health inequalities, particularly with higher prevalence of comorbidities in patients from more deprived areas.

### Strengths and limitations of the study

To the best of our knowledge, this is the largest study of comorbidities in patients with T2DM in England. The quality of the data is very high for our study period, primarily due to data recording in line with the QOF and the financial incentives offered to UK primary care for the management of chronic and other conditions such as T2DM.

However, the study has limitations. First, due to the low prevalence of some conditions in general and in specific groups, some comorbidities were excluded from the cluster analysis for all or some strata. However, all conditions were included in the frequency analysis which provides a starting point for the analysis of grouping patterns of specific conditions. Second, we selected only 18 conditions for which recording quality was high, but patients may have additional comorbidities impacting on their disease management and quality of life. Third, some of these comorbidities, like CKD and CHD, are closely linked to T2DM, to the extent of them being considered its complications. However, the majority of patients with these conditions do not have T2DM, while the characterisation of these conditions is immaterial to our analyses. Fourth, to identify patients with depression, we used an algorithm analysing prescriptions as well as diagnostic codes. We were unable to discriminate uses of antidepressants for other conditions such as obsessive-compulsive or bipolar disorders; therefore, patients with other mental health conditions might have been incorporated into the depression group. Fifth, the predictions of future prevalence rates were obtained from linear regression models, which are dependent on certain assumptions such as the linearity of the trend. Sixth, some of the conditions we modelled may be present but undiagnosed in our cohort. Seventh, for the hierarchical clustering, each comorbidity is necessarily considered into a single cluster, which may not be the case [21]. Last, some diagnostic criteria were also changed during the study period, for example, the diagnostic criteria for hypertension. Therefore, the average number of comorbidities calculated in our sample is likely to be underestimated both due to the finite set of conditions we used and to non-diagnosis in practice.

### Comparison with existing literature

We found that almost 75% of patients had at least one additional comorbidity at the time of T2DM diagnosis and 44% had at least two comorbidities. Prevalence of multiple conditions in addition to T2DM was lower than that reported in some clinical trials (90%) [22] or studies using administrative data (91.4%) [23] (84.6%) [24] but higher than in others (44%) [25]. However, our population was younger than in some studies, and we analysed a large but not exhaustive list of conditions. As expected, the burden of comorbidity increased with age, however, contrary to previous research [4, 8], which found a higher age-standardised prevalence of coexisting comorbidities in males or no gender difference, we found that the burden was higher in females. This reflects the pattern in the general population which shows that females tend to have more comorbid conditions than males [26]. This difference may relate to the surveillance bias with females being more likely to visit a general practitioner and therefore have a recorded diagnosis of comorbidity. In addition, previous studies tend to focus on conditions regarded as diabetes-concordant such as cardiovascular diseases and CKD [4]. Females with T2DM were found to have a lower probability of these having conditions and a higher prevalence of depression, which we included in our study [23]. The presence of mental health problems may have a significant impact on the ability of the patient to manage their condition, progression of T2DM [8, 16, 18]. Our findings of the high and increasing prevalence of depression in patients with T2DM imply that the inclusion of mental health conditions is essential in studies of comorbidities in this population. We found that the prevalence of all conditions except asthma and depression increased after diagnosis of T2DM. The fall in the prevalence of treated asthma during the follow-up may be related to the correlation between metformin use and decrease in asthma exacerbation [27]. Knowing that T2DM is highly correlated with obesity, as is asthma [28] and depression [29], it may be that patients after being diagnosed with T2DM work towards lowering their BMI, and therefore, both conditions may be resolved.

We observed a higher burden of comorbidity among people from the most deprived than the most affluent areas. Differences were also observed in the prevalence of specific conditions, notably higher prevalence of depression, CHD, asthma and COPD among people from the most deprived areas. This is consistent with other studies and may be explained by the higher prevalence of risk factors such as smoking, obesity and alcohol consumption [30, 31].

We found a very large increase in the prevalence of T2DM-comorbid depression, which is expected to rise over the next 10 years. The rising prevalence of depression and the large gender gap has also been observed for the general population [32]. There is an ongoing discussion over whether antidepressants are overprescribed [33, 34] which could explain the rise in depression observed in our analysis. Furthermore, the data may represent rises in conditions other than depression such as chronic pain for which antidepressants can be prescribed [35]. Although this discussion is inconclusive, the rise in antidepressant use in patients with T2DM should be a concern, with some evidence proposing that some antidepressants may be an independent risk factor for T2DM [36], suggesting that both conditions share similar risk factors. More research is needed to provide further insight into the increase in depression and antidepressants use in patients with T2DM. Nevertheless, people with both T2DM and depression may require tailored approaches of treatment for both conditions as depression was found to impair patients’ ability to manage their diabetes [15].

The observed and predicted stable or decreasing prevalence of comorbidities other than depression at the time of T2DM diagnosis may reflect the increase in the proportion of people diagnosed at a relatively early age [37]. This could mean that people are diagnosed with T2DM before they develop other comorbidities.

Our hierarchical clustering analysis showed that conditions regarded as diabetes-concordant (stroke, atrial fibrillation, CKD, CHD, hypertension, PVD and heart failure) tend to group together in all analysed groups. Cancer has been linked with different condition groups, depending on the analysed stratum. This may be due to the fact that we grouped all types of cancer into one condition. However, specific types of cancer may be more prevalent in different groups and be linked with the conditions sharing common risk factors. At the time of the T2DM diagnosis, the clusters seem to follow an expected pattern with lung diseases (asthma and COPD), mental health conditions (depression and SMI) and vascular conditions (PVD, CHD, stroke, atrial fibrillation and heart failure) grouped together. However, the grouping becomes more complex after the diagnosis with conditions needing different treatment and management likely to occur together. These complexities highlight the need for patient-centred approach. Furthermore, greater emphasis is needed on preventative actions and constant monitoring for conditions not closely related to the ones already experienced by the patient.

## Conclusion

Most people with T2DM have at least one other condition that can influence the self-management of diabetes and its progression. We found a high prevalence of T2DM-concordant conditions such as hypertension, CHD and CKD as well as T2DM-discordant conditions such as COPD and depression. The complexity of needs, specific to the patients’ comorbidities patterns as well as socio-economic situation, has to be considered when developing and providing comprehensive and precise care for people with T2DM. With the growing prevalence of T2DM [38], these complexities have to be taken into account when planning future care services, particularly given the higher cost of treating people with multiple conditions [39] and the lead times for developing appropriately skilled multi-disciplinary care teams. Further research is needed to identify the best course of action for treating people with multiple conditions, as recent research shows that existing interventions are not particularly effective for improving quality of life [40, 41].

Our analysis shows that cardiovascular conditions may become less prevalent among people with T2DM; however, clinicians will have to identify and manage the rising burden of comorbid mental health problems. Currently, services targeting people with T2DM are geared towards cardiovascular conditions. The growing burden of mental health conditions will require the restructuring of the services and workforce planning.

The cluster analysis showed that certain diseases are more likely to occur together; however, the specific grouping depends on the time after T2DM diagnosis. Further research could explore how individual patients experience the progression from no comorbidities to groups of conditions affecting different parts of the body and needing complex treatments.

## Additional files

Additional file 1: Definition of conditions being present; flow chart describing study cohort selection; additional figures S2-S18. (DOCX 4106 kb)

## Data Availability

The data that support the findings of this study are available from the UK CPRD, but restrictions apply to the availability of these data, which were used under licence for the current study, and so are not publicly available. The data are, however, available from the authors upon reasonable request and with permission of the UK CPRD.

## Abbreviations

CHD: Coronary heart disease
CKD: Chronic kidney disease
COPD: Chronic obstructive pulmonary disease
CPRD: Clinical Practice Research Datalink
IMD: Index of Multiple Deprivation
PVD: Peripheral vascular disease
QOF: Quality and Outcomes Framework
SMI: Severe mental illness
T2DM: Type 2 diabetes
