# Correction to: The comorbidity burden of type 2 diabetes mellitus: patterns, clusters and predictions from a large English primary care cohort

**DOI:** 10.1186/s12916-020-1492-5

**Published:** 2020-01-25

**Authors:** Magdalena Nowakowska, Salwa S. Zghebi, Darren M. Ashcroft, Iain Buchan, Carolyn Chew-Graham, Tim Holt, Christian Mallen, Harm Van Marwijk, Niels Peek, Rafael Perera-Salazar, David Reeves, Martin K. Rutter, Stephen F. Weng, Nadeem Qureshi, Mamas A. Mamas, Evangelos Kontopantelis

**Affiliations:** 1 NIHR School for Primary Care Research, Centre for Primary Care and Health Services Research, Manchester Academic Health Science Centre (MAHSC), University of Manchester, 5th floor Williamson Building, Manchester, M13 9PL UK; 2 Division of Population Health, Health Services Research & Primary Care, School of Health Sciences, Faculty of Biology, Medicine and Health, Manchester Academic Health Science Centre (MAHSC), University of Manchester, 5th floor Williamson Building, Manchester, M13 9PL UK; 3 Division of Pharmacy and Optometry, School of Health Sciences, Faculty of Biology, Medicine and Health, Manchester Academic Health Science Centre (MAHSC), University of Manchester, Manchester, M13 9PL UK; 4 NIHR Greater Manchester Patient Safety Translational Research Centre, University of Manchester, Manchester, M13 9PL UK; 5 Division of Informatics, Imaging, and Data Science, School of Health Sciences, Faculty of Biology, Medicine and Health, Manchester Academic Health Science Centre (MAHSC), University of Manchester, Manchester, M13 9PL UK; 6 Department of Public Health and Policy, Institute of Population Health Sciences, University of Liverpool, Liverpool, L69 3BX UK; 7 Research Institute for Primary Care and Health Sciences, Faculty of Medicine and Health Sciences, Keele University, DJW 1.54a, Staffordshire, ST5 5BJ UK; 8 Nuffield Department of Primary Care Health Sciences, University of Oxford, Oxford, OX2 6GG UK; 9 Division of Primary Care and Public Health, Brighton and Sussex Medical School, University of Sussex, Brighton, BN1 9PH UK; 10 NIHR Manchester Biomedical Research Centre, Manchester Academic Health Science Centre (MAHSC), University of Manchester, Manchester, M13 9PL UK; 11 NIHR Oxford Biomedical Research Centre, Oxford University Hospitals NHS Foundation Trust, Oxford, UK; 12 Centre for Biostatistics, Division of Population Health, Health Services Research and Primary Care, School of Health Sciences, Faculty of Biology, Medicine and Health, University of Manchester, Manchester, M13 9PL UK; 13 Division of Diabetes, Endocrinology and Gastroenterology, Faculty of Medicine, Biology and Health, University of Manchester, Manchester, M13 9PL UK; 14 Manchester Diabetes Centre, Manchester Academic Health Science Centre, Manchester University NHS Foundation Trust, Manchester, M13 0JE UK; 15 Primary Care Stratified Medicine (PRISM), Division of Primary Care, School of Medicine, University of Nottingham, Nottingham, NG7 2RD UK; 16 Keele Cardiovascular Research Group, Centre for Prognosis Research, Institute for Primary Care and Health Sciences, Keele University, Stoke-on-Trent, ST4 7QB UK

**Correction to: BMC Med (2019) 17:145**

**https://doi.org/10.1186/s12916-019-1373**

The original article [1] contains an omitted grant acknowledgement and affiliation as relates to the contribution of co-author, Rafael Perera-Salazar. As such, the following two amendments should apply to the original article:

1) Rafael Perera-Salazar should also be affiliated to **NIHR Oxford Biomedical Research Centre, Oxford University Hospitals NHS Foundation Trust** (as shown in the affiliations of this Correction article).

2) The following Acknowledgement statement should take precedence over that in the original article:
