# Supplementary material for: The comorbidity burden of type 2 diabetes mellitus: patterns, clusters and predictions from a large English primary care cohort
Source: BMC Med. 2019 Jul 25;17:145. doi: 10.1186/s12916-019-1373-y (PMC6659216; doi:10.1186/s12916-019-1373-y)
Supplement: Supplementary file 1 — Definition of conditions being present; flow chart describing study cohort selection; additional figures S2-S18. (DOCX 4106 kb) [file 12916_2019_1373_MOESM1_ESM.docx]

Additional file

| **Table S1: Definition of condition being present** | |
| --- | --- |
| **Comorbidity:** | **Definition:** |
| AF | Read code recorded ever |
| Asthma | Read code recorded ever AND any prescription in last 12 months |
| Cancer | First Read code recorded in last 5 years |
| CHD | Read code recorded ever |
| CKD | Read code recorded ever |
| COPD | Read code recorded ever |
| Dementia | Read code recorded ever |
| Depression | Read code in last 12 months OR ≥4 antidepressant prescriptions in last 12 months |
| Epilepsy | Read code recorded ever AND antiepileptic prescription in last 12 months |
| Hypertension | Read code recorded ever |
| Hypothyroidism | Read code recorded ever |
| Learning disability | Read code recorded ever |
| Osteoarthritis | Read code recorded ever |
| Osteoporosis | Read code recorded ever |
| SMI | Read code recorded ever |
| Stroke | Read code recorded ever |

| **Figure S1: Flow chart describing study cohort selection** |
| --- |
| **Patients diagnosed with T2DM between 1st April 2007 and 31st March 2017 from up to standard practices and of acceptable data quality.**  **190,058**  **112,318**  **Patients with link to HES, ONS and IMD data**  **4,034 patients under 35**  **2,991 patients with Read code for T1DM**  **3,548 patients registered for less than 365 days**  **171 patients with T2DM diagnosis recorded after the recorded date of death or transfer out of practice**  **2 patients with indeterminate gender**  **62 patients with missing IMD data**  **102,394**  **FINAL SAMPLE** |

*Abbreviations: T2DM – Type-2 Diabetes Mellitus, HES – Hospital Episode Statistics, ONS – Office for National Statistics, IMD – Index of Multiple Deprivation, T1DM – Type-1 Diabetes Mellitus*

| **Figure S2: Age-standardised prevalence of chronic conditions among females and males with T2DM from the least and most deprived areas at the time of T2DM diagnosis**  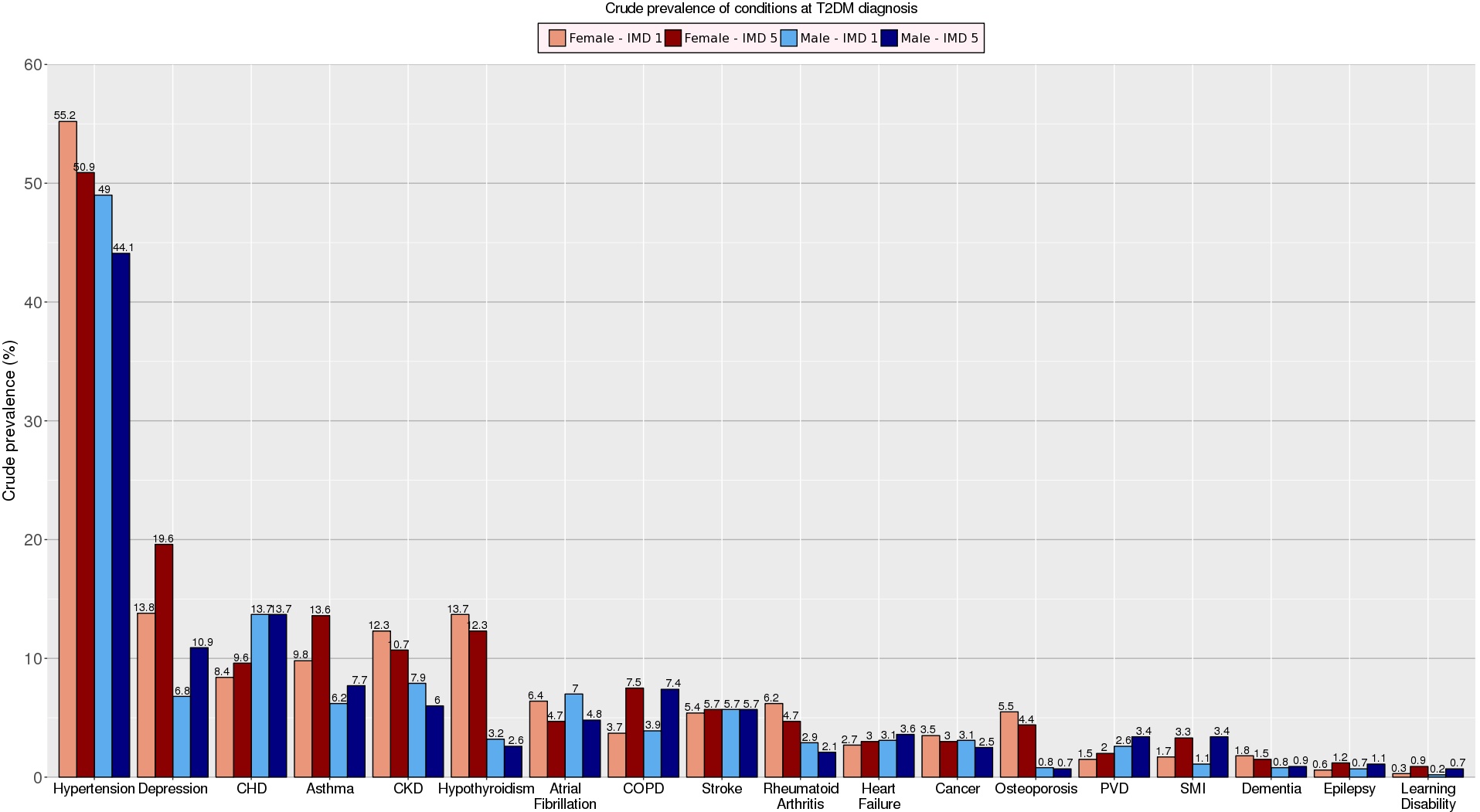 |
| --- |
|  |
| IMD – Index of Multiple Deprivation; CHD – coronary heart disease; CKD - chronic kidney disease; COPD - chronic obstructive pulmonary disease; PVD – peripheral vascular disease; SMI – severe mental illness |

| **Figure S3: Age-standardised prevalence of chronic conditions among females and males with T2DM from the least and most deprived areas two, five and nine years after T2DM diagnosis** |
| --- |
| 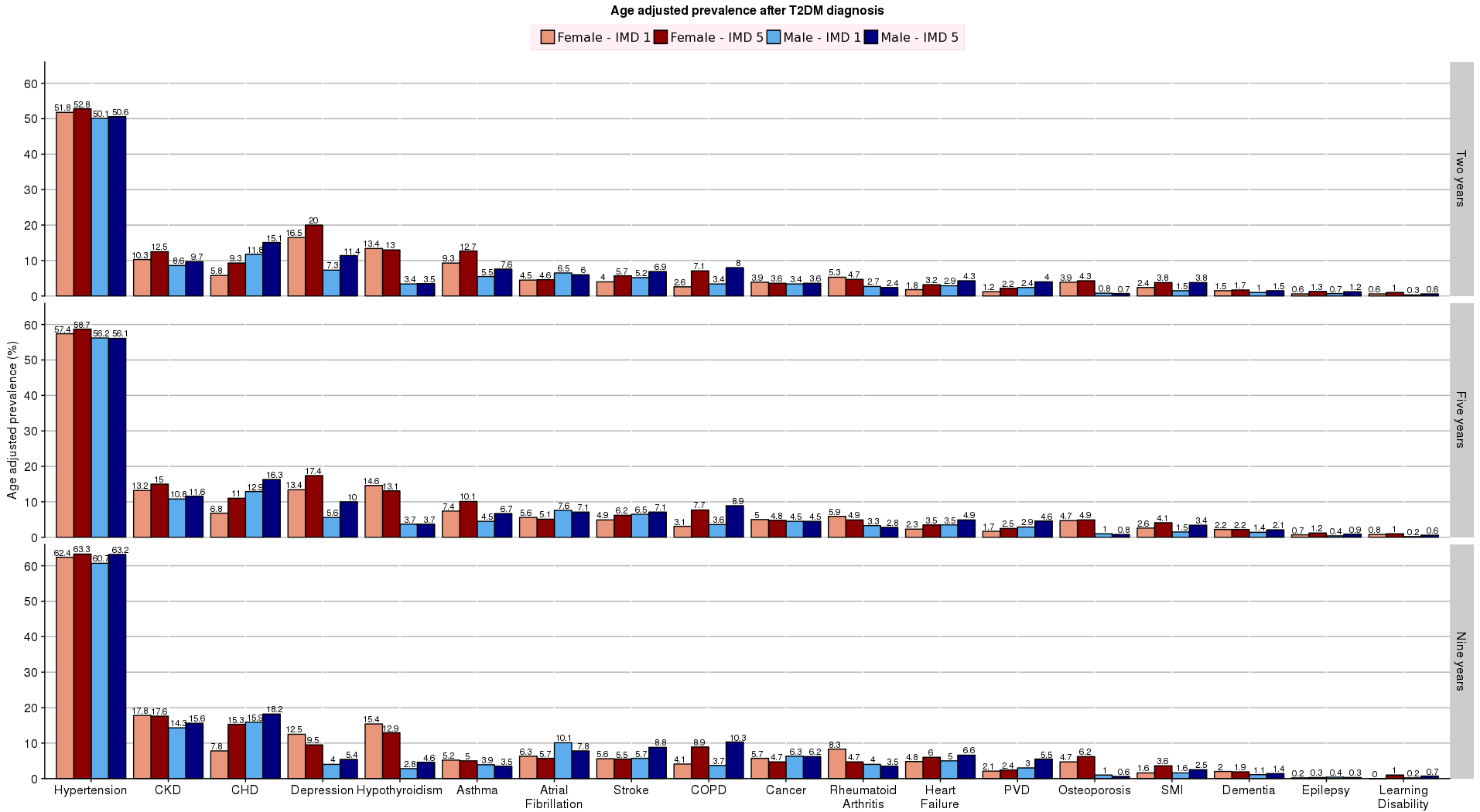 |
| IMD – Index of Multiple Deprivation; CHD – coronary heart disease; CKD - chronic kidney disease; COPD - chronic obstructive pulmonary disease; PVD – peripheral vascular disease; SMI – severe mental illness |

| **Figure S4: Crude and age-standardised co-prevalence of chronic conditions among people with T2DM at the time of T2DM diagnosis and two, five and nine years after.** | | | |
| --- | --- | --- | --- |
| 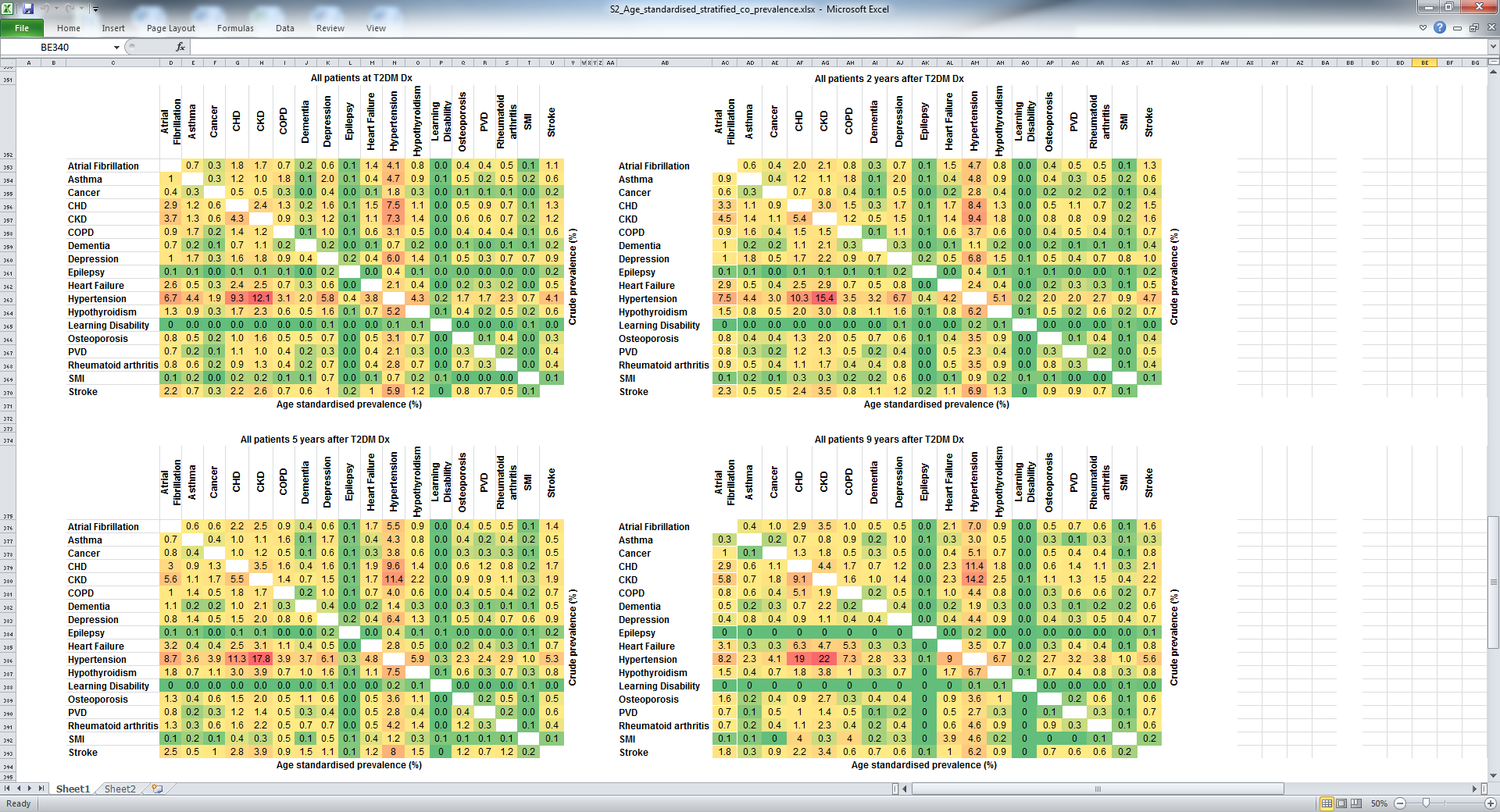 | | **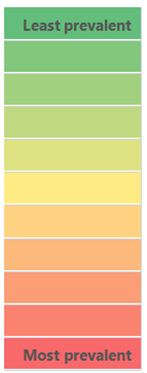** | |
| T2DM – type-2 diabetes mellitus; IMD – Index of Multiple Deprivation; Dx - diagnosis; CHD – coronary heart disease; CKD - chronic kidney disease; COPD - chronic obstructive pulmonary disease; PVD – peripheral vascular disease; SMI – severe mental illness | |  |  |

| **Figure S5: Age standardised and crude co-prevalence for people from the most deprived areas at the time of T2DM diagnosis and two, five and nine years after the diagnosis.** | |
| --- | --- |
| 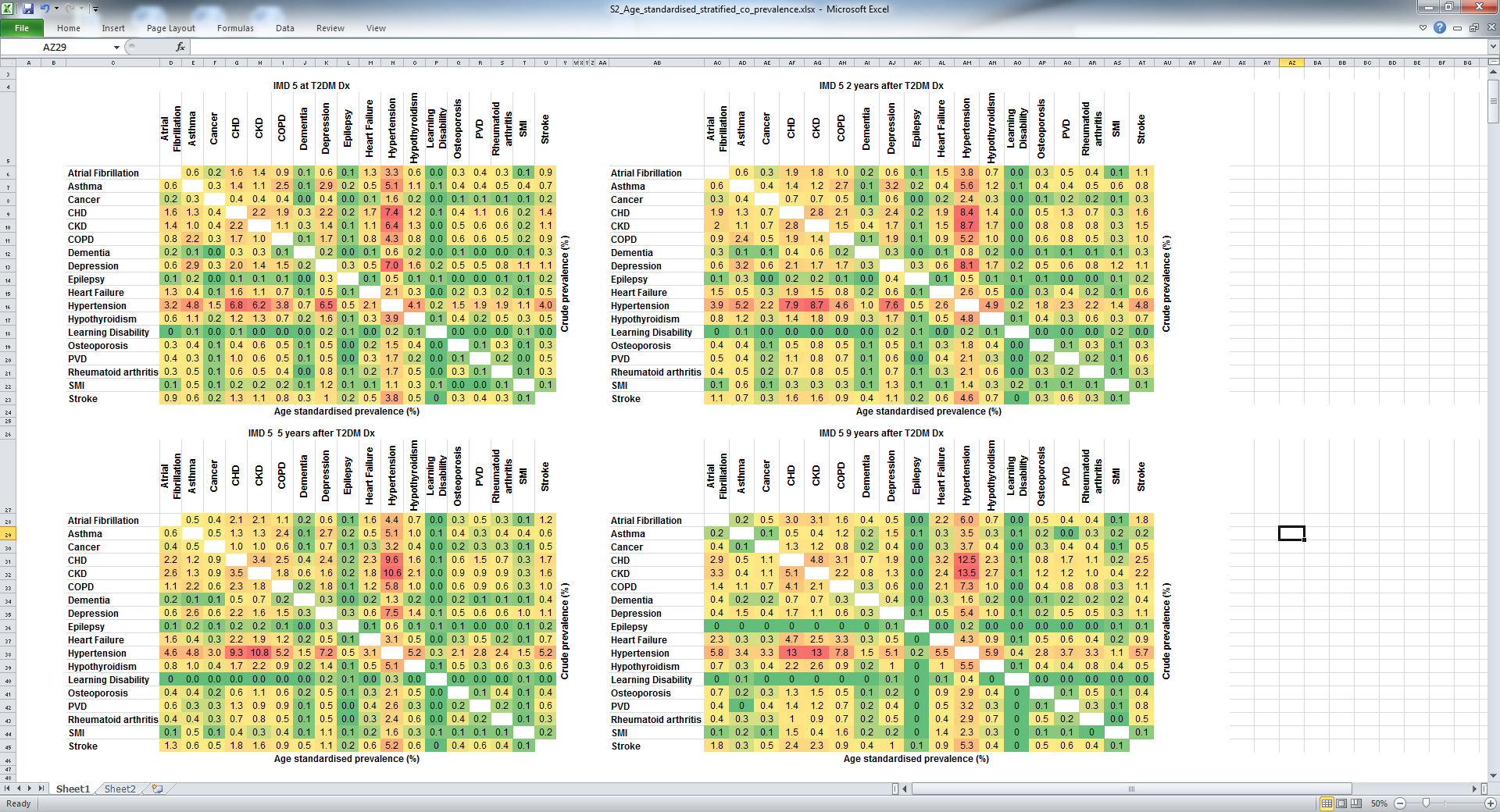 | **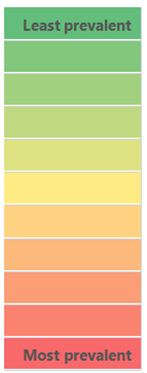** |
| T2DM – type-2 diabetes mellitus; IMD – Index of Multiple Deprivation; Dx - diagnosis; CHD – coronary heart disease; CKD - chronic kidney disease; COPD - chronic obstructive pulmonary disease; PVD – peripheral vascular disease; SMI – severe mental illness | |

| **Figure S6: Age standardised and crude co-prevalence for people from the least deprived areas at the time of T2DM diagnosis and two, five and nine years after the diagnosis.** | |
| --- | --- |
| 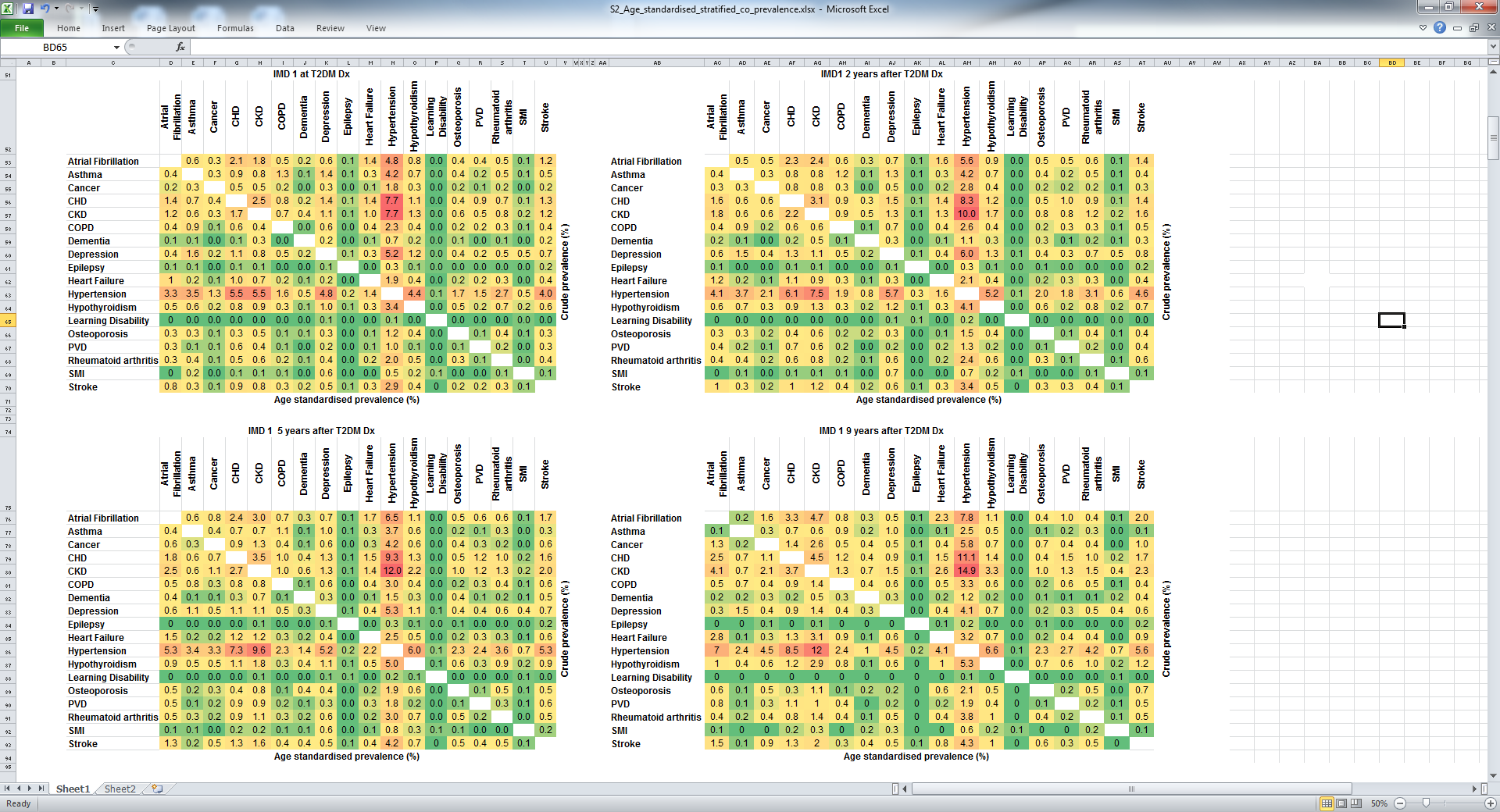 | **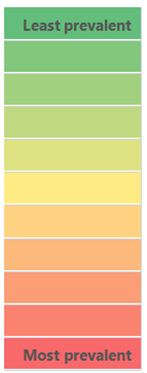** |
| T2DM – type-2 diabetes mellitus; IMD – Index of Multiple Deprivation; Dx - diagnosis; CHD – coronary heart disease; CKD - chronic kidney disease; COPD - chronic obstructive pulmonary disease; PVD – peripheral vascular disease; SMI – severe mental illness | |

| **Figure S7: Age standardised and crude co-prevalence for males at the time of T2DM diagnosis and two, five and nine years after the diagnosis.** | |
| --- | --- |
| 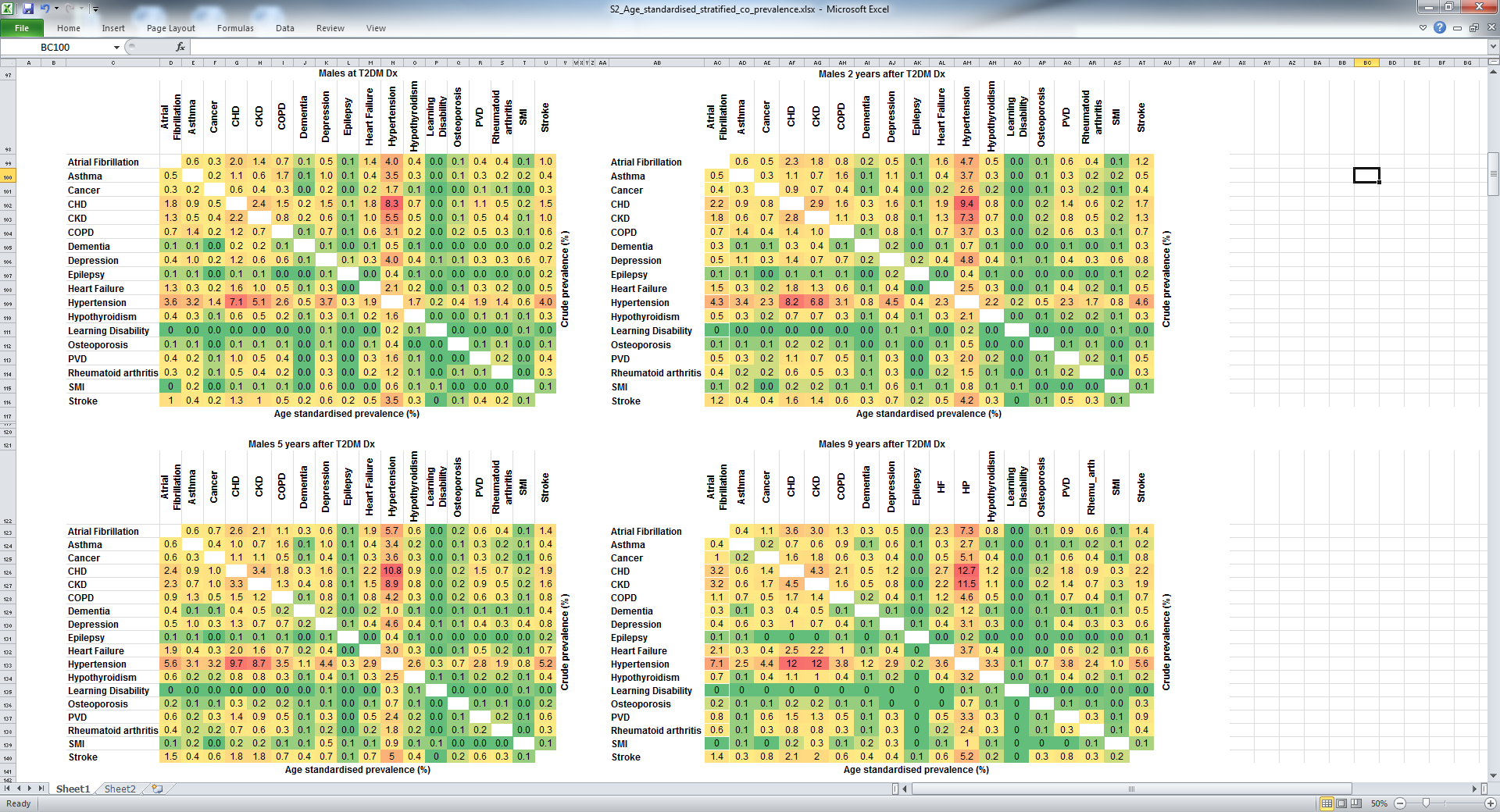 | **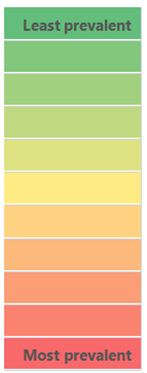** |
| T2DM – type-2 diabetes mellitus; Dx - diagnosis; CHD – coronary heart disease; CKD - chronic kidney disease; COPD - chronic obstructive pulmonary disease; PVD – peripheral vascular disease; SMI – severe mental illness | |

| **Figure S8: Age standardised and crude co-prevalence for females at the time of T2DM diagnosis and two, five and nine years after the diagnosis.** | |
| --- | --- |
| 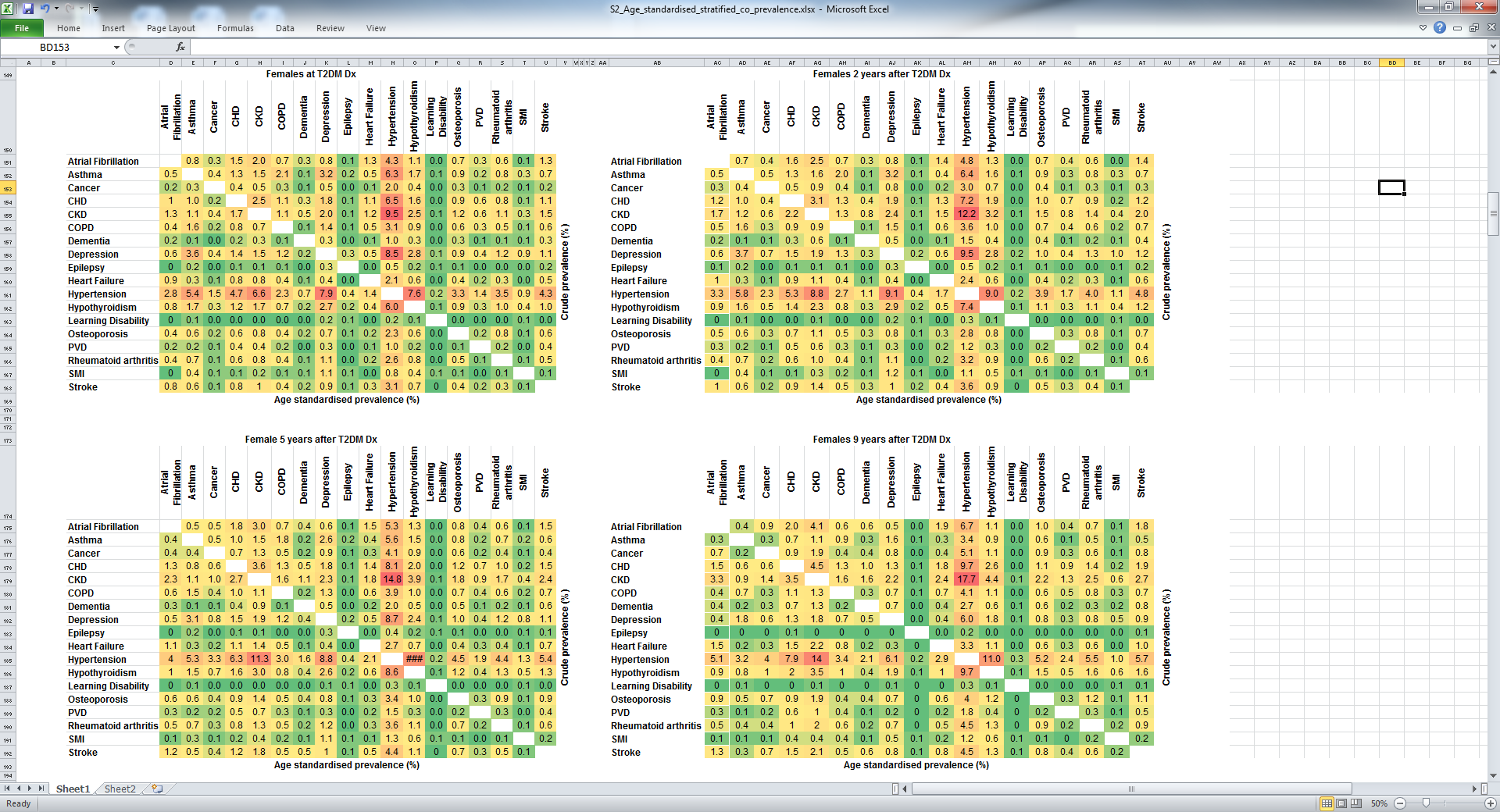 | **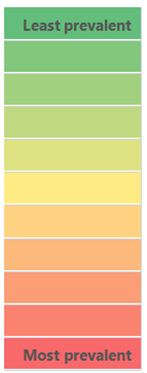** |
| T2DM – type-2 diabetes mellitus; Dx - diagnosis; CHD – coronary heart disease; CKD - chronic kidney disease; COPD - chronic obstructive pulmonary disease; PVD – peripheral vascular disease; SMI – severe mental illness | |

| **Figure S9: Age standardised and crude co-prevalence for people aged 75 and older at the time of T2DM diagnosis and two, five and nine years after the diagnosis.** | |
| --- | --- |
| 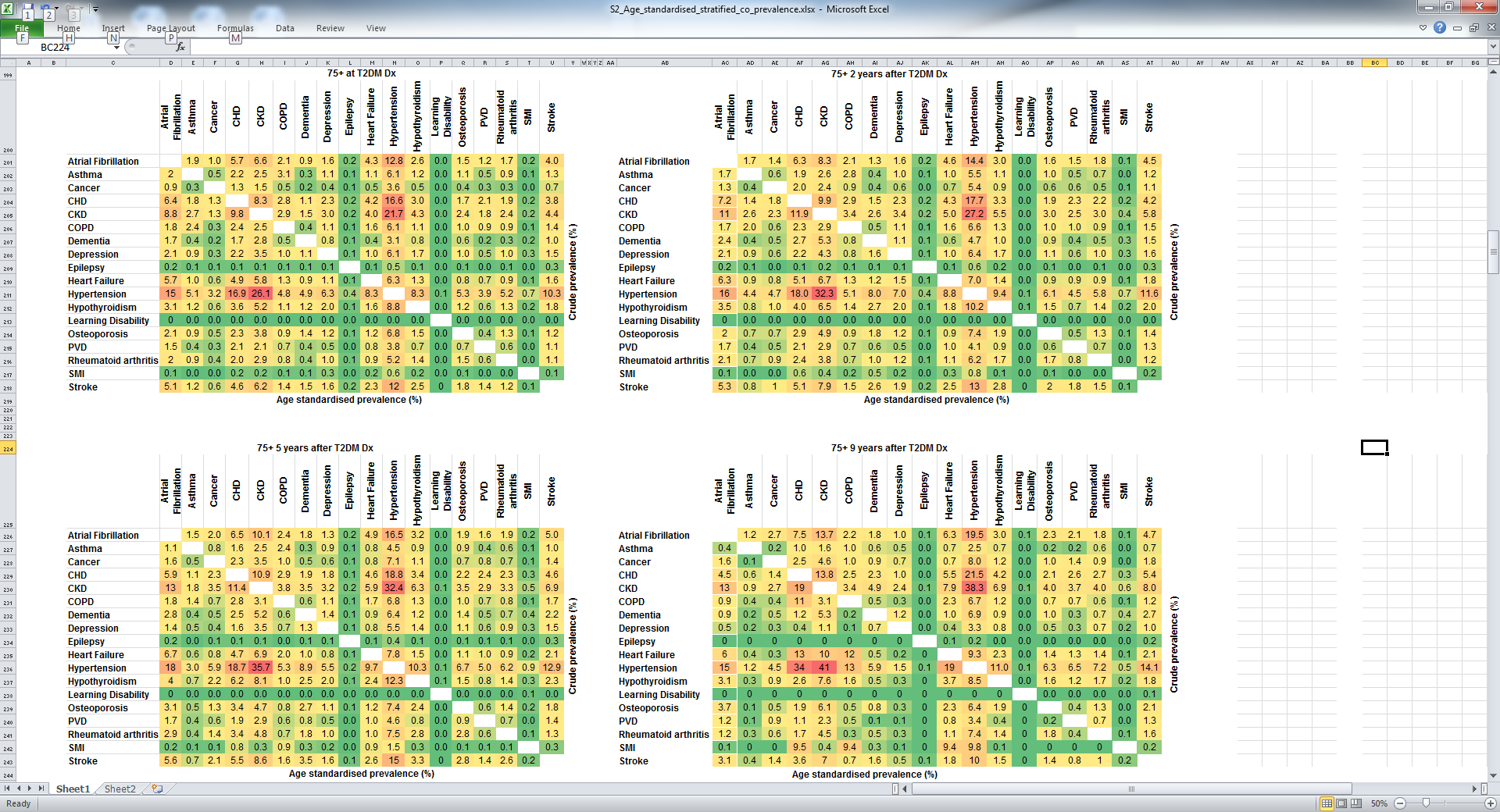 | **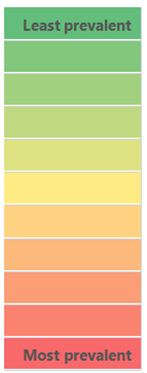** |
| T2DM – type-2 diabetes mellitus; Dx - diagnosis; CHD – coronary heart disease; CKD - chronic kidney disease; COPD - chronic obstructive pulmonary disease; PVD – peripheral vascular disease; SMI – severe mental illness | |

| **Figure S10: Age standardised and crude co-prevalence for people aged 55 to 74 at the time of T2DM diagnosis and two, five and nine years after the diagnosis.** | |
| --- | --- |
| 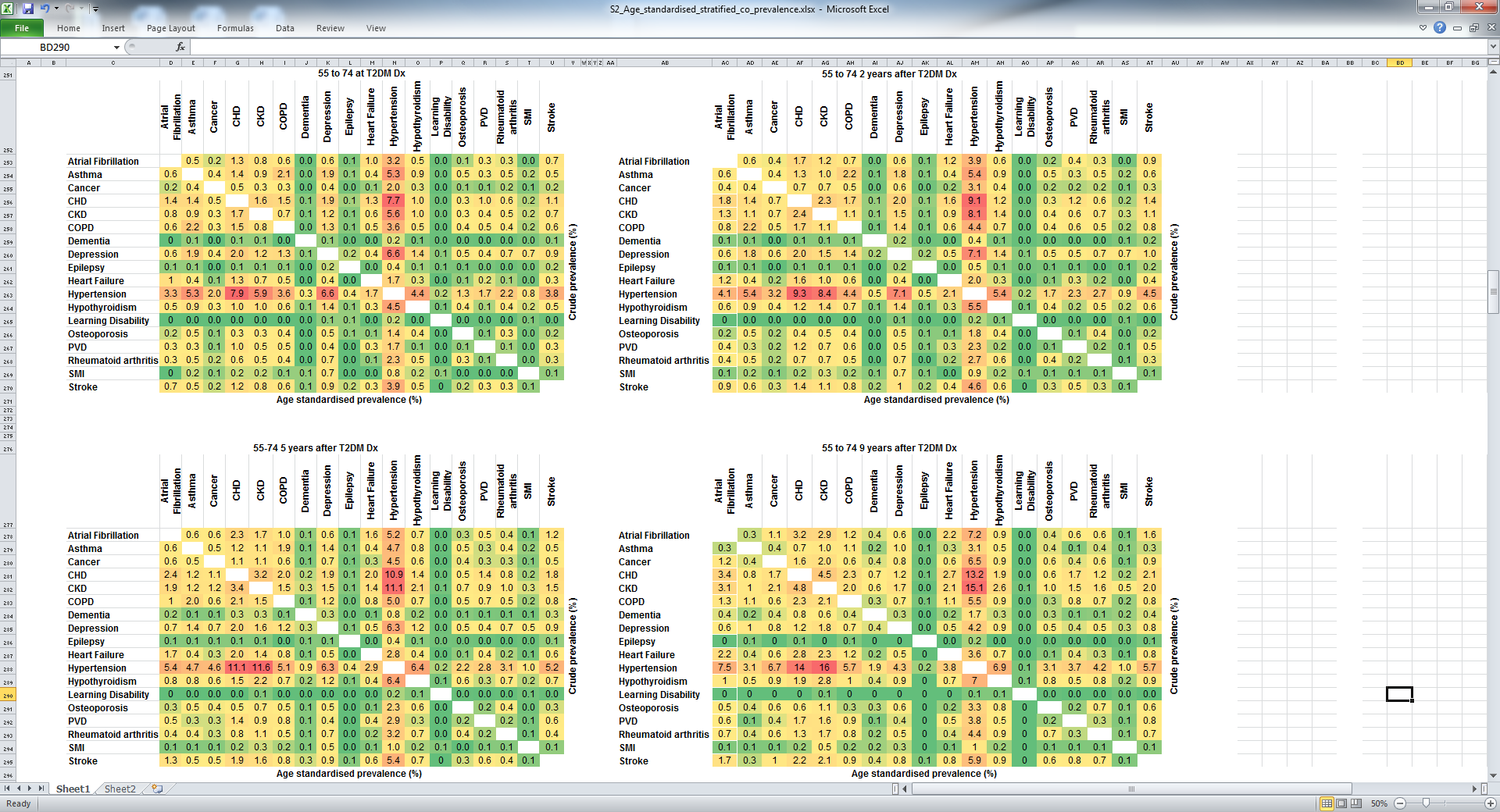 | **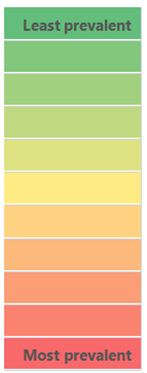** |
| T2DM – type-2 diabetes mellitus; Dx - diagnosis; CHD – coronary heart disease; CKD - chronic kidney disease; COPD - chronic obstructive pulmonary disease; PVD – peripheral vascular disease; SMI – severe mental illness | |

| **Figure S11: Age standardised and crude co-prevalence for people aged 35 to 54 at the time of T2DM diagnosis and two, five and nine years after the diagnosis.** | |
| --- | --- |
| 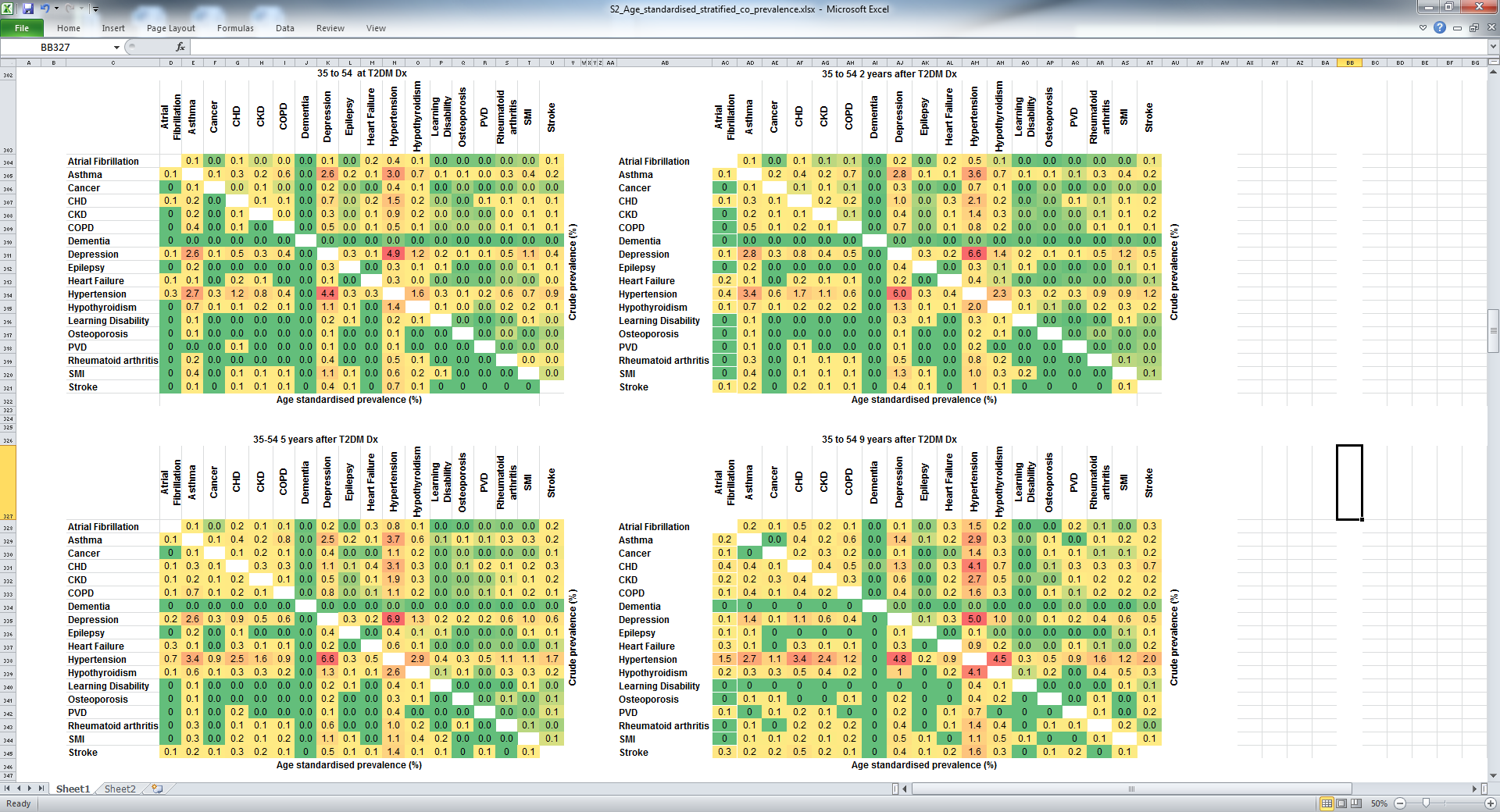 | **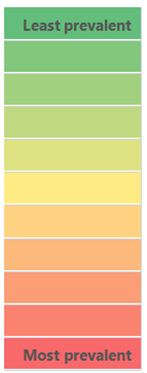** |
| T2DM – type-2 diabetes mellitus; Dx - diagnosis; CHD – coronary heart disease; CKD - chronic kidney disease; COPD - chronic obstructive pulmonary disease; PVD – peripheral vascular disease; SMI – severe mental illness | |

| **Figure S12: Cluster analysis of comorbidities for females at the time of T2DM diagnosis and two, five and nine years after.** |
| --- |
| 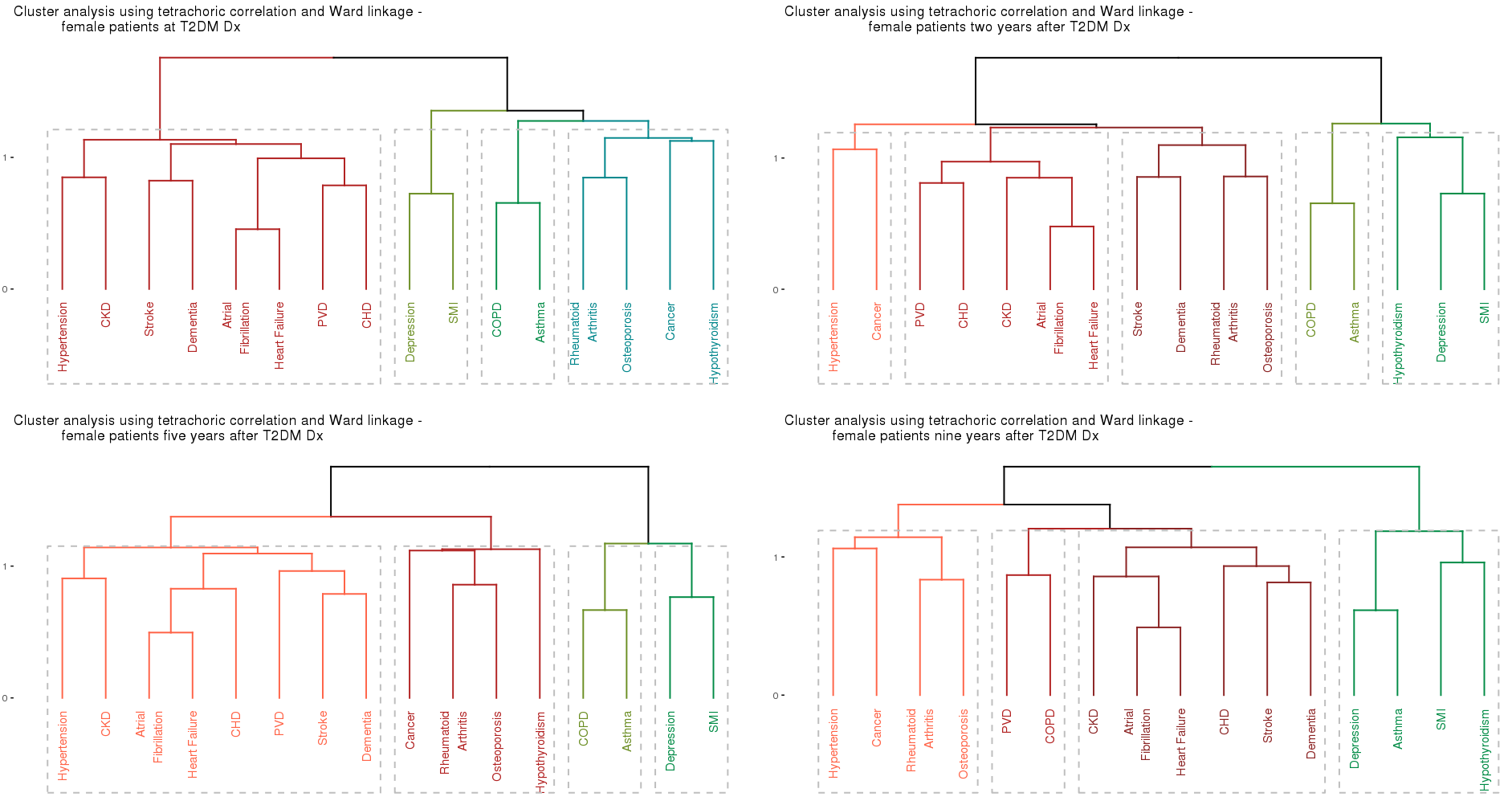 |
| CHD – coronary heart disease; CKD - chronic kidney disease; COPD - chronic obstructive pulmonary disease; PVD – peripheral vascular disease; SMI – severe mental illness |

| **Figure S13: Cluster analysis of comorbidities for males at the time of T2DM diagnosis and two, five and nine years after.** |
| --- |
| 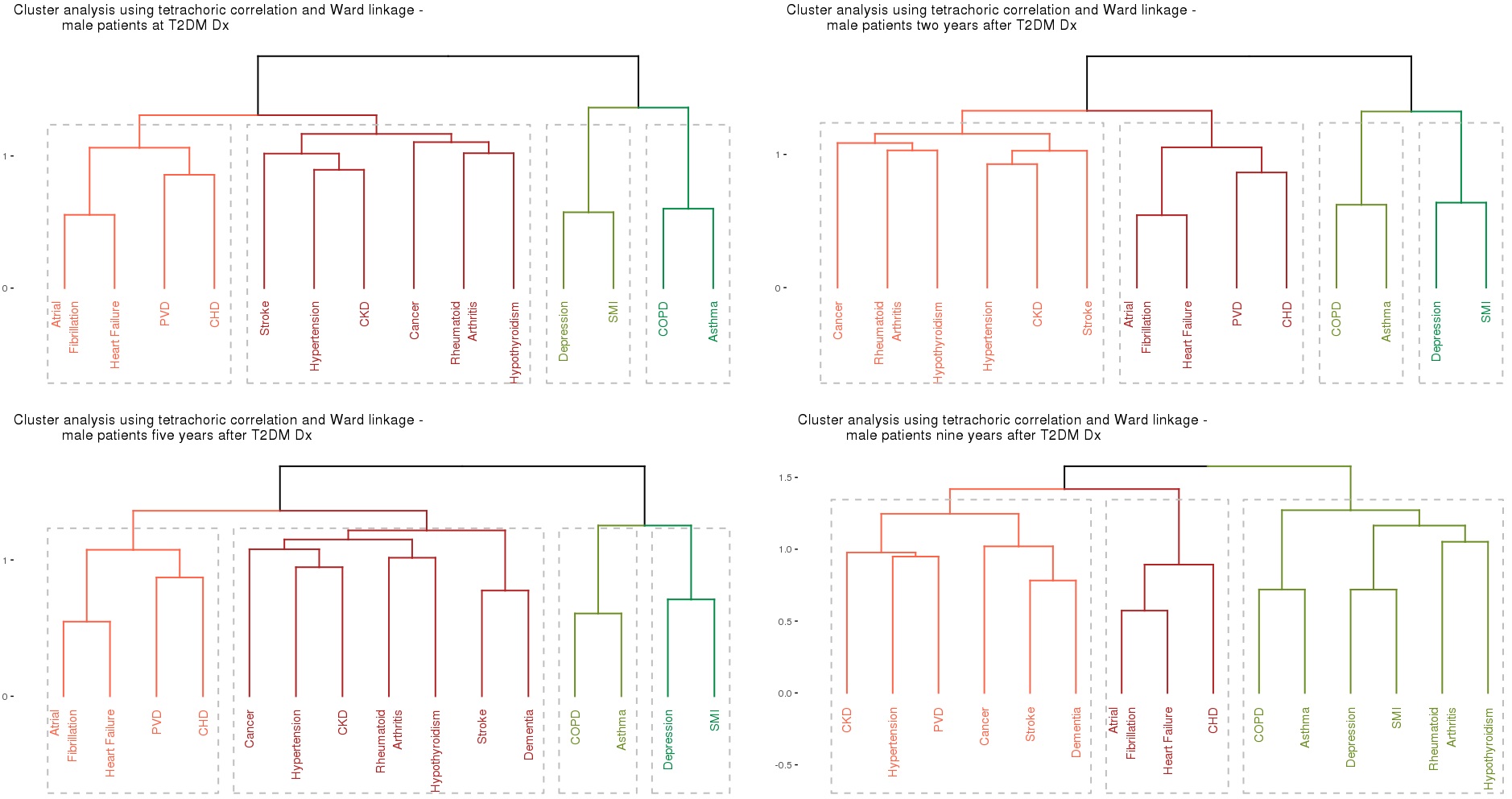 |
| CHD – coronary heart disease; CKD - chronic kidney disease; COPD - chronic obstructive pulmonary disease; PVD – peripheral vascular disease; SMI – severe mental illness |

| **Figure S14: Cluster analysis of comorbidities for people from least deprived areas at the time of T2DM diagnosis and two, five and nine years after.** |
| --- |
| 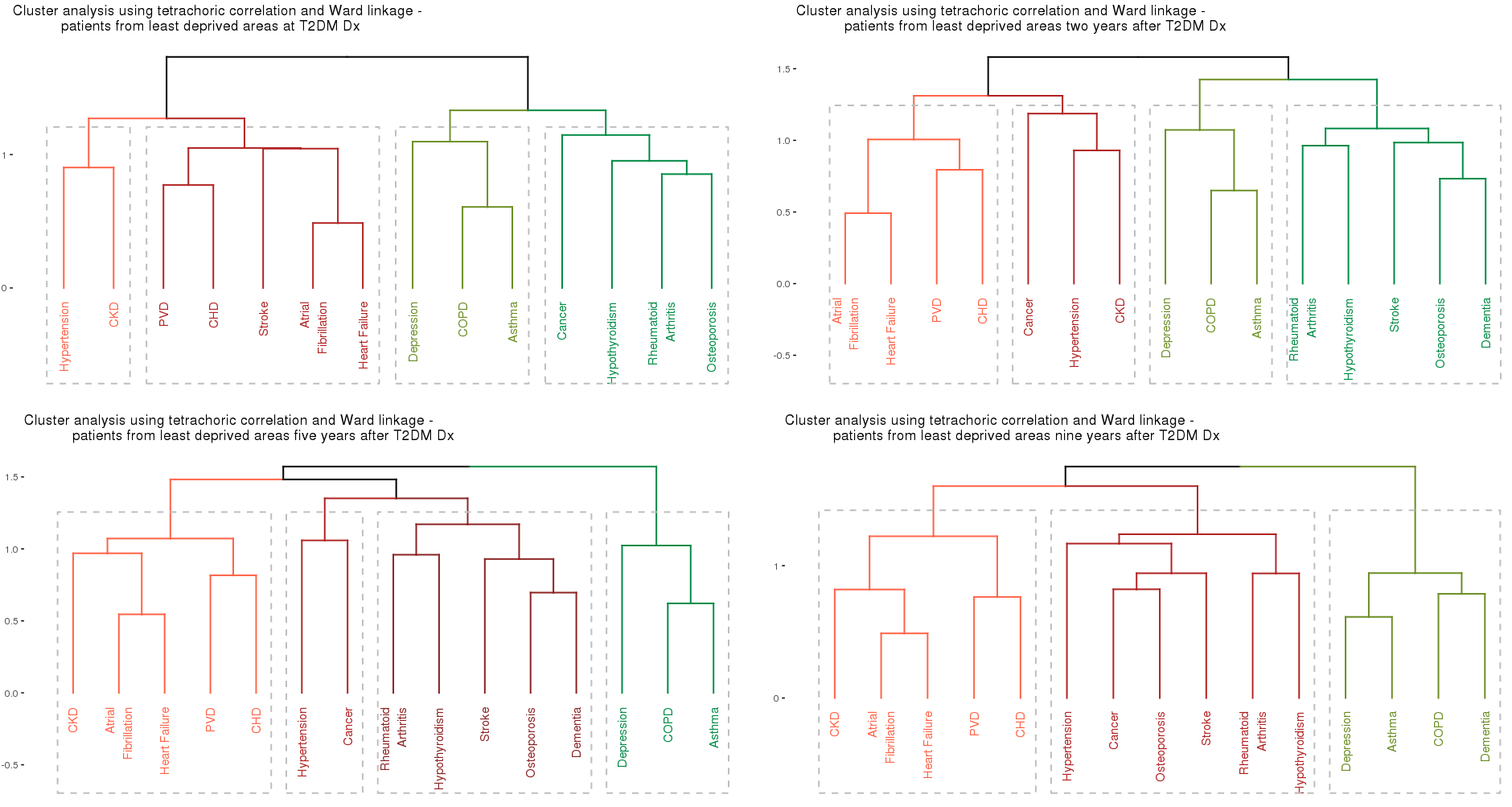 |
| CHD – coronary heart disease; CKD - chronic kidney disease; COPD - chronic obstructive pulmonary disease; PVD – peripheral vascular disease; SMI – severe mental illness |

| **Figure S15: Cluster analysis of comorbidities for people from most deprived areas at the time of T2DM diagnosis and two, five and nine years after.** |
| --- |
| 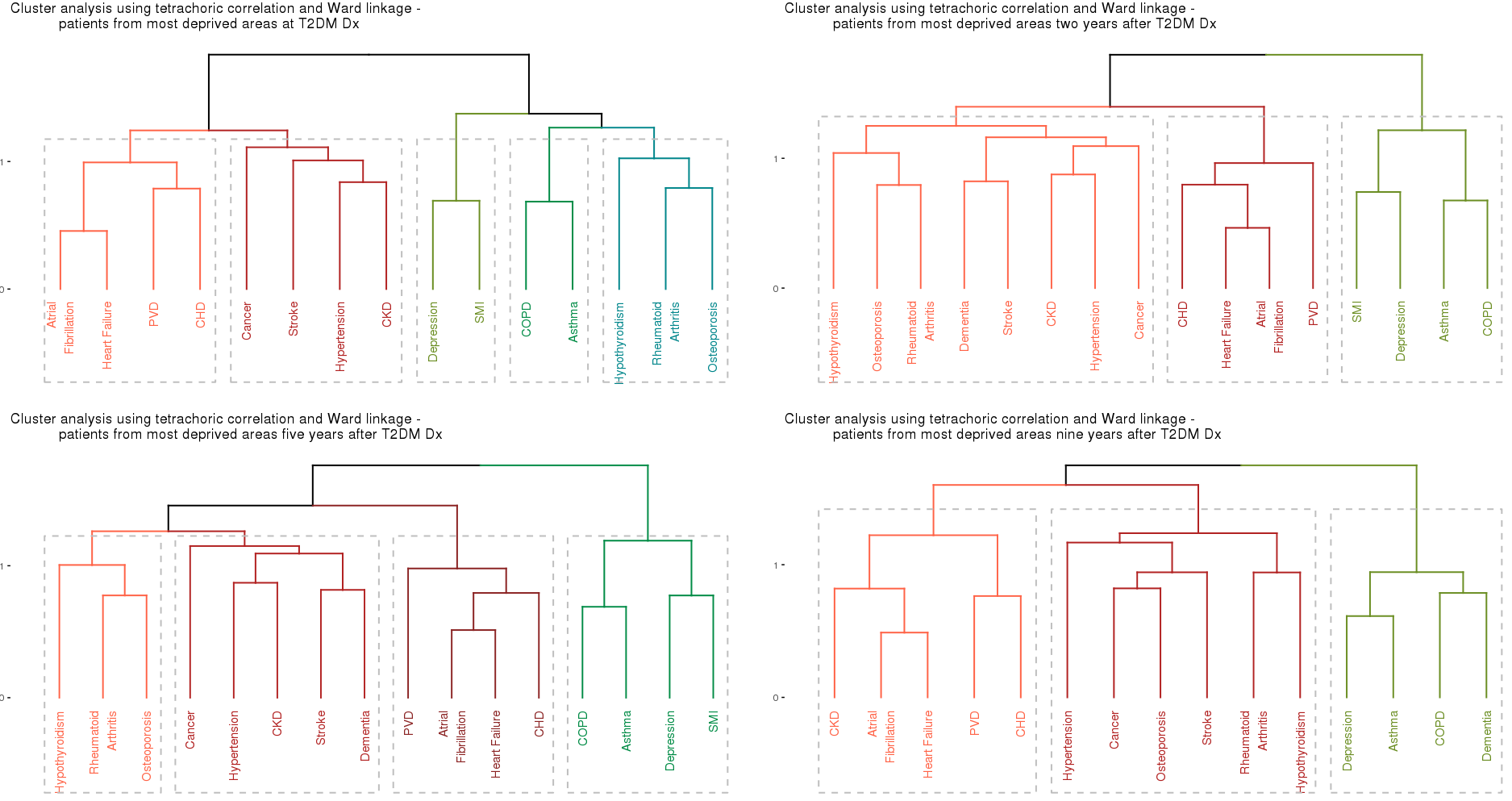 |
| CHD – coronary heart disease; CKD - chronic kidney disease; COPD - chronic obstructive pulmonary disease; PVD – peripheral vascular disease; SMI – severe mental illness |

| **Figure S16: Cluster analysis of comorbidities for people age 35 to 54 at the time of T2DM diagnosis and two, five and nine years after.** |
| --- |
| 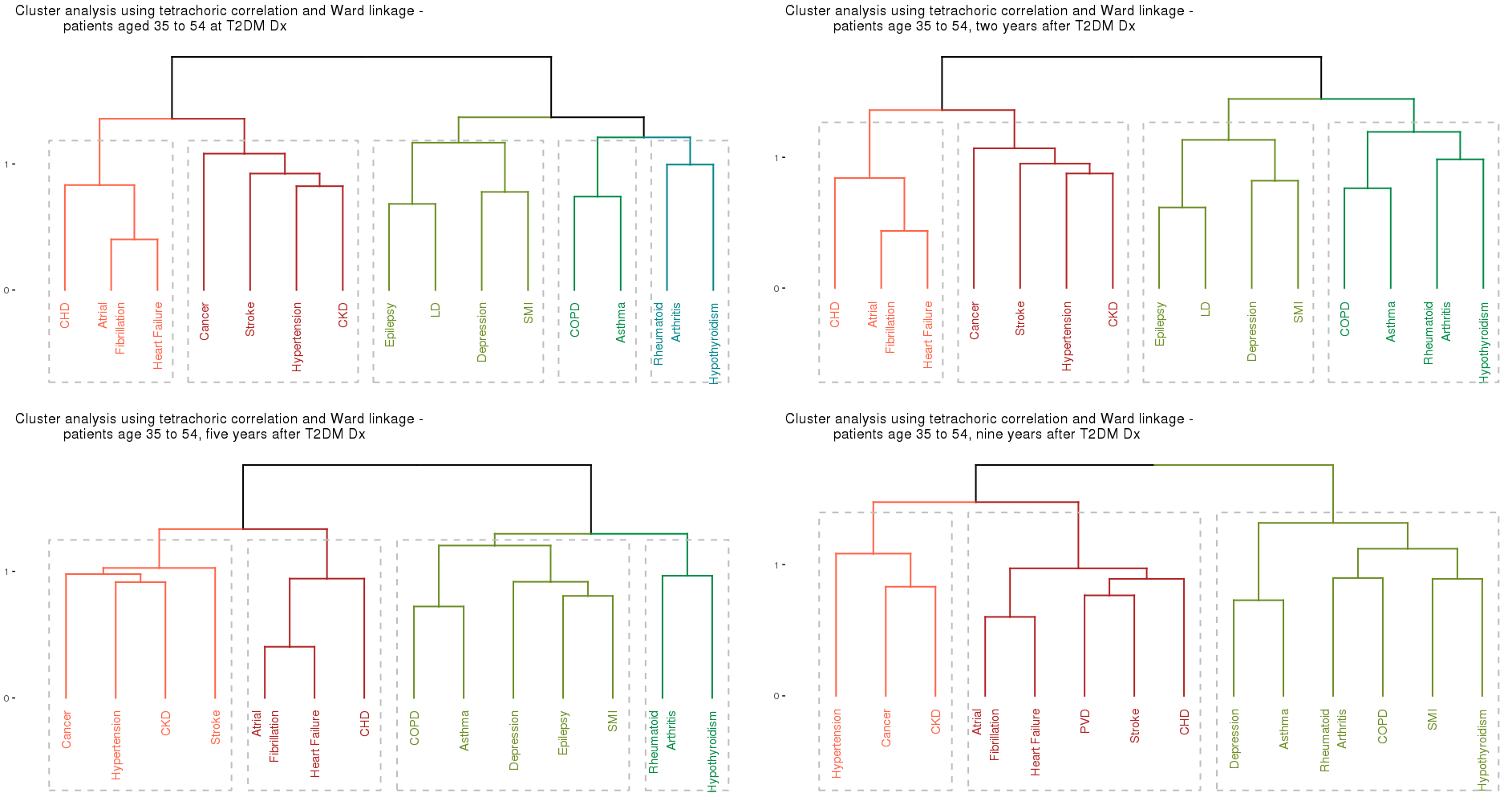 |
| CHD – coronary heart disease; CKD - chronic kidney disease; COPD - chronic obstructive pulmonary disease; PVD – peripheral vascular disease; SMI – severe mental illness |

| **Figure S17: Cluster analysis of comorbidities for people age 55 to 74 at the time of T2DM diagnosis and two, five and nine years after.** |
| --- |
| 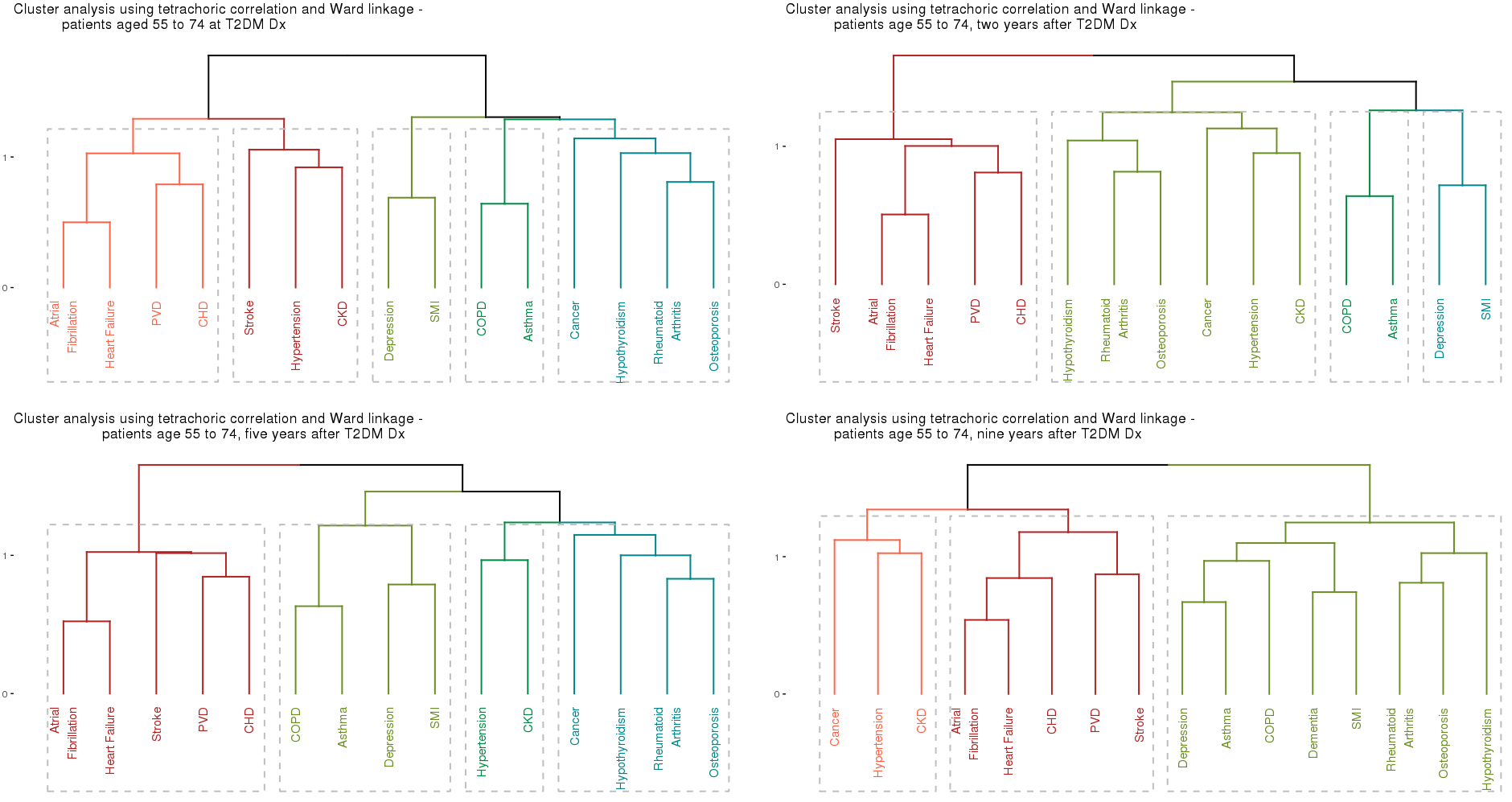 |
| CHD – coronary heart disease; CKD - chronic kidney disease; COPD - chronic obstructive pulmonary disease; PVD – peripheral vascular disease; SMI – severe mental illness |

| **Figure S18: Cluster analysis of comorbidities for people age 74 and older at the time of T2DM diagnosis and two, five and nine years after.** |
| --- |
| 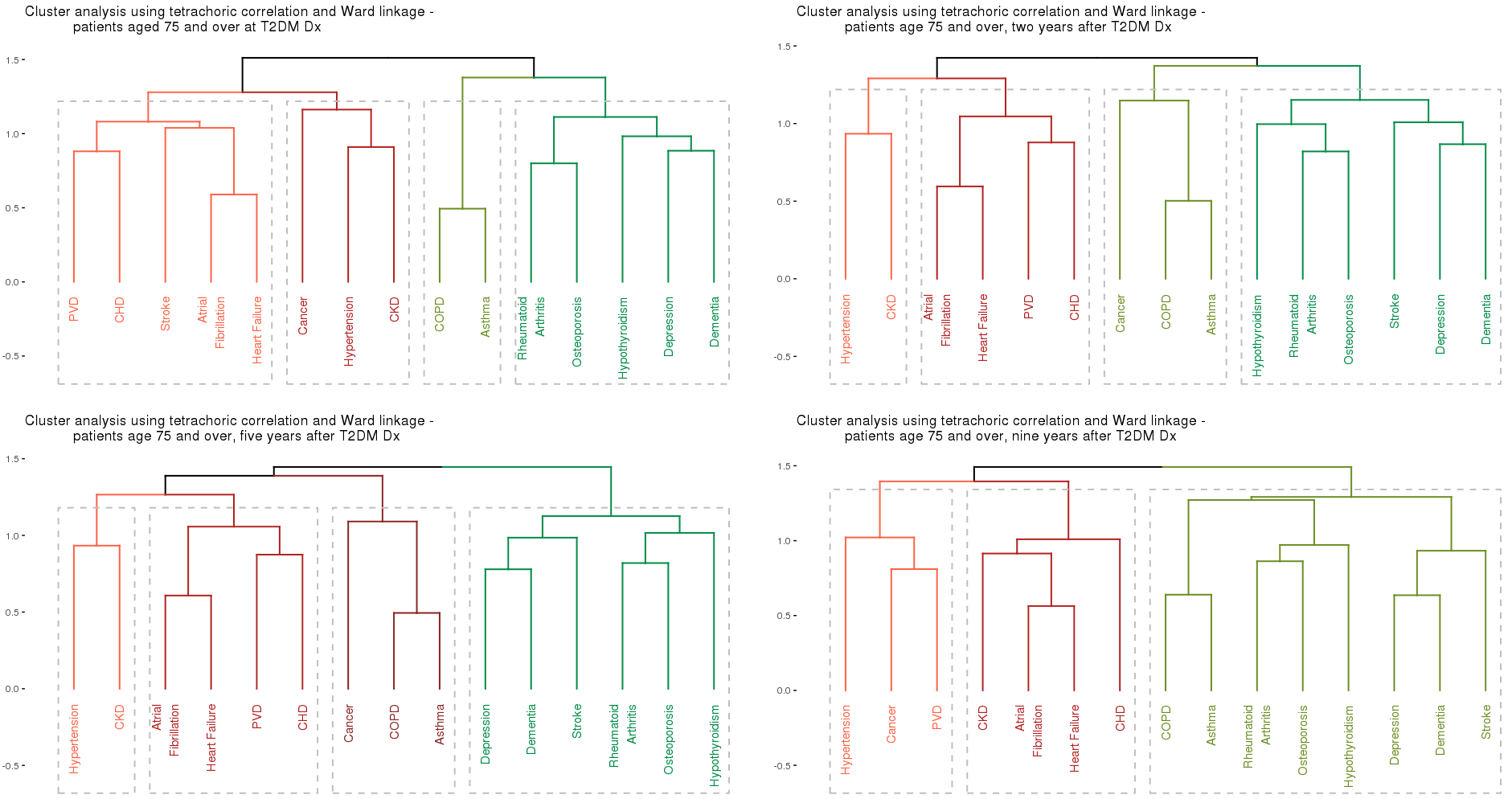 |
| CHD – coronary heart disease; CKD - chronic kidney disease; COPD - chronic obstructive pulmonary disease; PVD – peripheral vascular disease; SMI – severe mental illness |
